# Supplementary material for: Study protocol for Hear Me Read (HMR): A prospective clinical trial assessing a digital storybook intervention for young children who are deaf or hard of hearing
Source: PLoS One. 2024 May 31;19(5):e0302734. doi: 10.1371/journal.pone.0302734 (PMC11142545; doi:10.1371/journal.pone.0302734)
Supplement: S2 File — (DOCX) [file pone.0302734.s002.docx]

**PROTOCOL TITLE:**

HRP-503 – Hear Me Read 2021 Clinical Trial

**PRINCIPAL INVESTIGATOR:**

Prasanth Pattisapu, MD

Pediatric Otolaryngology (ENT)

Center for Surgical Outcomes Research, Abigail Wexner Research Institute

**VERSION NUMBER/DATE: 7, 03/07/2023**

HRP-503 – Hear Me Read 2021 Trial v7

**REVISION HISTORY**

| **Version #** | **Revision #** | **Version Date** | **Summary of Changes** | **Consent Change?** |
| --- | --- | --- | --- | --- |
| 2 | 1 | 3/01/2022 | Updated eligibility criteria, removed EOWPVT Assessment | N |
| 3 | 2 | 4/28/2022 | Updated recruitment methods | N |
| 4 | 3 | 6/20/2022 | Minor text clarifications | N |
| 5 | 4 | 9/21/2022 | Change reflects addition of clinical document to caregiver packet | N |
| 6 | 5 | 12/19/2022 | Change PI name and contact information | Y |
| 7 | 6 | 03/07/2023 | Updated study procedures | Y |

**Table of Contents**

# Study Summary

# Objectives

# Background

# Study Endpoints

# Study Intervention

# Procedures Involved

# Data and Specimen Banking*

# Sharing of Results with Subjects*

# Study Timelines*

# Inclusion and Exclusion Criteria*

# Vulnerable Populations*

# Local Number of Subjects

# Recruitment Methods

# Withdrawal of Subjects*

# Risks to Subjects*

# Potential Benefits to Subjects*

# Data Management* and Confidentiality

# Provisions to Monitor the Data to Ensure the Safety of Subjects*

# Provisions to Protect the Privacy Interests of Subjects

# Compensation for Research-Related Injury

# Economic Burden to Subjects

# Consent Process

# Process to Document Consent in Writing

# Setting

# Resources Available

# *Multi-Site Research – N/A*

# ****Protected Health Information Recording****

# References

# Study Summary

| **Study Title** | “Effectiveness of adding a novel digital storybook intervention platform in therapy to improve speech, language, and literacy outcomes in children with hearing loss” |
| --- | --- |
| **Study Design** | Prospective clinical trial |
| **Primary Objective** | Determine if in-person speech-language therapy (SLT) with a novel digital storybook intervention (Hear Me Read, HMR) improves **vocabulary, speech and language outcomes** in young children who are D/HH compared with in-person therapy alone. |
| **Secondary Objective(s)** | Determine if in-person speech-language therapy (SLT) with a novel digital storybook intervention (Hear Me Read, HMR) improves **literacy outcomes** in young children who are D/HH compared with in-person therapy alone. |
| **Research Intervention(s)/ Investigational Agent(s)** | Hear Me Read! is an IOS-based software application that enables parents/caregivers and treating speech-language pathologists to partner together to help deaf/hard of hearing children (D/HH) achieve reading, speech and language goals through interactive digital storybook reading |
| **IND/IDE #** | N/A |
| **Study Population** | Children seen in the Hearing Program at Nationwide Children’s Hospital who are deaf/hard of hearing, their parent/caregiver, and their treating Speech Language Pathologist (SLP).  **Child Eligibility:**   - ***Inclusion:***   - Male and female children ages 3.0-5.11 years at time of initial assessment and:     - Bilateral Sensorineural or mixed hearing loss at least (PTA≥30dB) ***or***     - Auditory neuropathy in both ears - ***Exclusion:***   - English not primary language   - Standard score CLS >2 SD from normal on CELF-P3   **Parent/Caregiver Eligibility:**   - ***Inclusion:***   - Parent/caregiver of eligible child (must be child’s legal guardian) - ***Exclusion:***   - None   **Treating SLP Eligibility:**   - ***Inclusion:***   - Treating therapist/SLP for participating child - ***Exclusion:***   - None |
| **Sample Size** | Approximately 50 children and their parents/caregivers, and the child’s treating SLP. |
| **Study Duration for individual participants** | 1 year |
| **Study Specific Abbreviations/ Definitions** | D/HH: D/deaf or hard of hearing  ASL: American Sign Language  AVT: Auditory-Verbal Therapy  HMR: Hear Me Read (name given to digital intervention)  PHL: Permanent Hearing Loss  MHL: Mixed Hearing Loss  SNHL: Sensorineural Hearing Loss  SLP: Speech-Language Pathologist  SLP_T_: Treating Speech-Language Pathologist  SLP_S_: Study Speech-Language Pathologist  SLT: Speech and Language Therapy  HA: Hearing Aid  CI: Cochlear Implant  ANSD: Auditory Neuropathy Spectrum Disorder  ROWPVT-4: Receptive One Word Picture Vocabulary Test CELF P3: Clinical Evaluation of Language Fundamentals Preschool-3  WS: Word Structure  SC: Sentence Comprehension  EV: Expressive Vocabulary  FD: Following Directions  RS: Recalling Sentences  BC: Basic Concepts  WC: Word Classes  CLS: Core Language Score  RLI: Receptive Language Index  ELI-lan: Expressive Language Index  PA: Phonological Awareness  PRS: Preliteracy Rating Scale  ELI-lit: Emerging Literacy Index  IRB: Institutional Review Board  ENT: Ear, Nose, Throat. Also known as Otolaryngology.  CRS: Clinical Research Services  BTO: Behavioral Trials Office |

# Background and Objectives

- 1. Describe the purpose, specific aims, or objectives of this study

Children who are D/deaf or hard of hearing (D/HH) face challenges in developing spoken language and literacy^1-2^. Quality therapy is needed for D/HH children to achieve speech, language, and literacy goals. Expert therapy resources are scarce, and families face challenges obtaining it. Novel digital therapeutic interventions may be able to improve and extend therapeutic benefits. Currently, no digital therapy interventions designed for D/HH children are available. Our research team has developed a novel prototype mobile app called Hear Me Read (HMR), designed to use digital stories as multimodal tools in therapy for speech, language, and literacy, in a platform that enhances family engagement for D/HH children.

The *objective of the proposed study* is to investigate the clinical efficacy of adding HMR to therapy in a population of D/HH children at Nationwide Children’s Hospital**. Our *central hypothesis* is that in-person speech-language therapy *with* a novel digital storybook intervention platform (HMR) *improves* vocabulary, speech and language outcomes in young children who are D/HH compared with in-person therapy alone.** In addition, this study will serve as a pilot clinical study to determine which specific outcomes are most sensitive to change with digital interventions, and which features are most useful for development and inform future, larger studies in this population.

**Specific Aim 1: Determine if in-person speech-language therapy *with* a novel digital storybook intervention (Hear Me Read) improves vocabulary, speech and language outcomes in young children who are D/HH compared with in-person therapy alone.**

**Specific Aim 2: Determine if in-person speech-language therapy *with* a novel digital storybook intervention (Hear Me Read) improves literacy outcomes in young children who are D/HH compared with in-person therapy alone.**

**Additional Aims:**

- Determine if in-person speech-language therapy *with* a novel digital storybook intervention (Hear Me Read) ***improves* therapy experience** *or* **book-reading habits** for families of young children who are deaf/hard of hearing (D/HH) compared with in-person therapy alone.
- Obtain feedback from parents/caregiver and treating SLPs about the design and usability of the Hear Me Read app.

We will measure these secondary outcomes using parent/caregiver and treating SLP questionnaires, and in-app metrics of app usage.

Our *long-term goal* is to use HMR to accelerate development of reading interventions, transform therapy for D/HH children and greatly increase availability of evidence-based and experience-driven therapy.

- 1. State the hypotheses to be tested.

**Hypothesis for Specific Aim 1**: In-person speech-language therapy with a novel digital storybook intervention (HMR) **improves** vocabulary, speech and language outcomes in young children who are D/HH compared with in-person therapy alone.

We will measure standardized, behavioral measures of vocabulary, speech, and language outcomes in D/HH children after 6 months first with in-person speech-language therapy alone (SLT-only), and again after another 6 months of in-person speech-language therapy with the digital storybook intervention (SLT+Digital).

**Hypothesis for Specific Aim 2:** In-person speech-language therapy with a novel digital storybook intervention (HMR) **improves** literacy outcomes in young children who are D/HH compared with in-person therapy alone.

We will measure standardized, behavioral measures of literacy outcomes in D/HH children after 6 months first with in-person speech-language therapy alone (SLT-only), and again after another 6 months of in-person speech-language therapy with the digital storybook intervention (SLT+Digital).

# Background

- 1. *Summarize the relevant prior research on this topic and gaps in current knowledge within the field of study*:

Nearly 3 per 1000 newborns are born with hearing loss, making this **one of the most common birth anomalies**^14^.

**Children who are D/HH (even in the mild to severe range) face challenges in developing spoken language and literacy, such as with semantics, vocabulary, learning new words and concepts, and syntax and reading**^1-2^. Historically, the reading skills of deaf children have been poor, with graduating teenager reading scores comparable with first- to fourth-grade reading level^3-5^. Even kids with access to sound with technology such as cochlear implants may have wide variability in language and literacy outcomes^16-18^. Young children who do not attain early literacy skills are at a higher risk long-term for academic and social problems^6-9^.

**D/HH children need frequent in-person therapy with a pediatric hearing loss expert to develop spoken language and literacy but face challenges in access**^19-20^. SLPs specializing in American Sign Language (ASL), or auditory-verbal therapy (AVT) are primarily located around larger cities or academic centers. Therapy sessions are currently in-person and can require visits as often as 1-2x/week, which challenges many parents/caregivers. Digital interventions potentially increase access.

**National literacy and education experts emphasize** **the importance of reading storybooks to young children to enhance literacy**^21-25^. Interactive reading strategies using dialogic reading techniques or meaning-related talk during reading can improve oral language development outcomes such as receptive vocabulary^26-28^. Vocabulary building strategies such as providing definitions and context, picture and video support, and multiple exposures of novel words are established^25^

**Current therapy approaches in D/HH children frequently use books as interventions.** The few published reading intervention studies in children who are D/HH indicate that shared or dialogic book reading using higher level facilitative language techniques can also improve oral language abilities and vocabulary^29-31^. The quality of the parent/caregiver-child interaction around stories is critical. The AVT approach routinely coaches and guides families to use books to practice scaffolding techniques to achieve listening and spoken language skills and reading comprehension^32^.

**Novel digital interventions present an opportunity to extend therapy for D/HH children.** Mobile phone/tablet technologies with digital electronic books are now commonplace in modern homes and schools. In the current COVID-19 pandemic era where many children have been unable to attend schools or therapy sessions in person, quality digital interventions are sought after. Digital technology may be able to serve as a method to extend educational resources to families in whom there are barriers to making therapy appointments, and who cannot otherwise obtain services. Currently, children from lower-income homes aged 0-8 years spend 2x as much time with screen media each day than those from higher-income homes. Black children and children from lower income households are more likely to read on digital devices.^10^

**Children who are D/HH have unique learning needs, such as using multiple modalities** – such as visual cues for speechreading – especially if they have limited access to sound^33-34^. This bimodal (audiovisual) fusion occurs in children even years after cochlear implantation^35^. Parents/caregivers of D/HH children often adapt their child-directed communication to include tactile (touch) and visual (facial expressions and gestures) modalities, in addition to vocal alterations^36^. Digital technologies may be able to uniquely provide multimodal input to D/HH children.

**D/HH children currently lack adequate vocabulary interventions.** D/HH children often have impoverished vocabularies, less ability to learn vocabulary incidentally (i.e. overhearing) and require more intentional instructional strategies to improve their overall lexicon^37-39^. Good expressive and receptive vocabulary is a key foundation in early literacy and language development, yet vocabulary interventions were found lacking in a review of the research in D/HH children^40^. At the same time, vocabulary interventions appear responsive to book reading interventions^30-31,41^. Other intervention studies have targeted literacy outcomes such as phonological awareness, fluency, reading comprehension^42^.

**Currently, there is little data regarding the efficacy and use of digital interventions targeting D/HH.** In a review of technology-based intervention studies in D/HH children, nearly all studies were of poor quality^45^. Digital reading interventions can vary from exact digital replications of print books to those embedded with instructions, assessments, audiovisual enhancements and prompts for human interaction. Digital books can potentially be harmful to the shared book reading experience if it increases distractions and reduces face-to-face interaction^11-12^. Thus, the design of educational apps must be done thoughtfully^44^ and *with evidence* to support effectiveness, especially in vulnerable kids. High-quality prospective studies such as randomized controlled trials of digital language and literacy interventions in D/HH children are nearly non-existent. *The only study*, Messier & Wood (2015), demonstrated feasibility of a parent/caregiver-implemented e-book reading intervention in 18 children with cochlear implants, and improved vocabulary gains compared with traditional book ***in just 6 weeks***^41^.

To address challenges faced by D/HH children, we have developed a novel mobile app called Hear Me Read (HMR). HMR is described in greater detail below. This study is a **prospective trial** investigating the effectiveness of adding a novel digital storybook intervention (Hear Me Read) to traditional in-person therapy on speech, language, and literacy outcomes in D/HH children.

- 1. Describe any relevant preliminary data

*Development of Hear Me Read*

Developed at NCH by our study team, Hear Me Read is an IOS-based software application that enables parents/caregivers and speech-language pathologists (SLP) to partner together to help deaf/hard of hearing children (D/HH) achieve reading, speech and language goals through interactive digital storybook reading. This platform is intended to be guided by a treating SLP to enhance targeted speech, language, and literacy goals derived from individualized, standard evaluations. In a therapist mode, a SLP can select from a library of high-quality, illustrated digital children’s stories. Stories can be selected based on auditory or grammatical features in the book such as reading level, or quantity of specific targets (such as first/last letter of word, part of speech, number of syllables, etc). A therapist can then select specific targets to be highlighted in the story (e.g. all words starting with “s”, and all “-ing” words) **(See Malhotra et al (2020)).** After selection, a book can be assigned to family, with personalized embedded prompts (“sticky notes”) that can guide families on appropriate comprehension questions to ask. These enhancements can be cleared and the same story selected again with different targets, allowing a story to be used for multiple goals over time. In parent/child mode, the family can select from assigned books, and read their story. Stories can be read with audio-visual features, therapy targets, clickable text, and illustrations turned on or off. A parent/caregiver can create personalized video recording of themselves reading the text, which is segmented, and can then be selected and played back within the text of the story to highlight audio and visual cues in a familiar voice. Families can review the targeted goals input by the SLP, as well as the interactive prompts. Vocabulary targets can be clicked to demonstrate audio and an image.

Hear Me Read can help families and therapists track reading progress and prescribe new reading assignments. In-app metrics can measure the time spent in the book, the number of times read, and progress within the book.

**Click for Video tutorial (Therapist Mode):**

<https://vimeo.com/637531824/e16bee0665> *(no password)*

**Click for Video (Features of Parent Mode):** <https://vimeo.com/522025931/49a8c6d0b2> *(password: HearMeRead)*

Preliminary evaluation of the Hear Me Read technology has been performed. Usability testing performed through semi-structured group interviews of parents/caregivers of D/HH children found the app to be pleasing and the functionalities useful; user feedback was then used for iterative improvements to design^46^. Additionally, 17 SLPs were surveyed using a structured survey, after using the HMR read app, using both open and close ended questions about the app was used to collect the information from the reviewers. The app was reviewed for use, understandability, special features, user interface, user experience and purposefulness. The survey also gave the reviewers an opportunity to write their perception on the advantages/ weaknesses and give feedback on the app. The information from the forms was then transferred verbatim to a Microsoft Word document and a qualitative analysis was done to summarize the major findings from the survey by the Behavioral Trials Office biostatistician at NCH. They found value in the technology, felt that it could benefit additional populations, and were generally enthusiastic, and recommended additional features. Their comments and feedback on user interface and functionality were incorporated into the currently updated version of the app.

*DeForte S, Sezgin E,* ***Huefner J, Lucius S, Luna J****, Satyapriya AA****, Malhotra P****. Usability of a Mobile App for Improving Literacy in Children With Hearing Impairment: Focus Group Study. JMIR Hum Factors. 2020 May 28;7(2):e16310. doi: 10.2196/16310. PMID: 32205305; PMCID: PMC7290449.*

- 1. Based on the existing literature, provide the scientific or scholarly rationale for and significance of your research and how will it add to existing knowledge

To address challenges faced by D/HH children, we have developed a novel mobile app called the Hear Me Read (HMR). In line with the recommendations by NAEYC, our intention with HMR is to use digital stories as multimodal therapy tools for speech, language, and literacy and to develop a platform for delivery that enhances family engagement for D/HH children. HMR is described in greater detail in Section 3.2. How well HMR, or other digital interventions, can achieve these goals is yet unknown.

This study is a **prospective clinical trial** investigating the effectiveness of adding a novel digital storybook intervention platform (Hear Me Read) to traditional in-person therapy on speech, language, and literacy outcomes in D/HH children.

The Hear Me Read platform is in its infancy, and we anticipate a future of great impact. Hear Me Read is a significant step towards developing evidence-based digital reading interventions for D/HH children. It is a platform that can incorporate changes quickly and evolve in iterations. The present study is focused on determining how well this technology is an extension of currently applied therapeutic principles. However, beyond simply extending current models of therapy, it is a flexible, programmable tool that will allow us to explore the underlying processes behind early literacy and language development in D/HH children of all ages, auditory abilities, and communication strategies. HMR can objectively measure components of digital interventions through in-app measures, determine which are most effective, and inform design of future ones. Our aspiration is to scale the technology to a cloud-based, widely available interface that can process ***any*** digital book for therapeutic purposes and accessibility in multiple modalities. In addition to providing vulnerable or isolated populations with greater access to enhanced storybook material, it can also harness the exponential power of big data, social media interactivity and feedback and enable parental/caregiver input to inform the design of future interventions (i.e. which books are most useful, what interventions are most useful). Additionally, we envision curricula and modules created by experts (using ASL, or AVT principles) to empower speech therapists who do not have D/HH expertise to work with children and confidently extend therapy. Hear Me Read has the potential to accelerate the development of reading interventions, transform therapy for D/HH children and greatly increase the availability of evidence-based and experience-driven therapy.

# Study Endpoints

- 1. Describe the primary and secondary study endpoints.

The primary study endpoints acquiring the primary study outcomes of standardized behavioral measures of speech, language, and literacy outcomes. These include Raw Score, Standard Score, Percentile Rank, Age Equivalent, and Growth Scale Values where applicable for the following tests: Receptive One Word Picture Vocabulary Test, Clinical Evaluation of Language Fundamentals Preschool-3 and subtests). The clinical standard test-retest interval for these behavioral measures in D/HH children is 6 months.

Secondary outcomes include measures of the impact on therapy experience, book engagement/book-reading habits, and feedback on the design and usability of the Hear Me Read app. These will be measured with parent/caregiver and SLP questionnaires, and in-app metrics of app usage.

Primary outcomes are measured pre-trial, at 6 months (after 1st intervention arm), and at 12 months (after 2nd intervention arm). Secondary outcomes are measured at 6 months (after 1st intervention arm), and at 12 months (after 2nd intervention arm). Study phase will end upon cessation of data collection after the 2nd intervention arm and participants’ participation.

The secondary endpoint will be the completion of data analysis, which concludes the management of PHI.

- 1. Describe any primary or secondary safety endpoints.

N/A, as this study poses no more than minimal risk to patients enrolled.

# Study Intervention/Investigational Agent

- 1. *Describe the study intervention and/or investigational agent (e.g., drug, device) that is being evaluated.*

Hear Me Read is an IOS-based software application that enables parents/caregivers and speech-language pathologists (SLP) to partner together to help deaf/hard of hearing children (D/HH) achieve reading, speech and language goals through interactive and multimodal digital storybook reading. **Please see detailed description of Hear Me Read in Section 3.2.**

This platform is intended to be guided by a treating SLP to enhance targeted speech, language, and literacy goals derived from individualized, standard evaluations. Hear Me Read has a therapist mode and parent mode.

Hear Me Read can help families and therapists track reading progress and prescribe new reading assignments. In-app metrics can measure the time spent in the book, the number of times read, and progress within the book.

Other study interventions (*ROWPVT-4, CELF-P3)* include age-appropriate behavioral surveys administered by a SLP to assess a child’s language and communication skills. These consist of verbal or pointing tasks in response to verbal or picture prompts and questions. In addition, electronic questionnaires and surveys (REDCap) regarding reading, therapy experience, and feedback on the HMR app will be given to parents/caregivers and treating SLPs.

- 1. Drug/Device Handling: If the research involves drugs or device, describe your plans to store, handle, and administer those drugs or devices so that they will be used only on subjects and be used only by authorized investigators.

No drugs will be given, nor medical devices will be used on patients.

NCH study iPads will be provided to intervention study children and their parent/caregiver directly, at first therapy visit when the HMR intervention is initiated with the participant. A reading/therapy app will be used as prescribed by a treating speech-language therapist. iPad serial numbers will be documented and stored. These iPads will be password protected, requiring input password before access to any apps. Passwords will be provided only to treating therapists, investigators, study staff, and to the study family. When not in use and upon completion of the study, investigators will store the iPads in a locked drawer, in a locked office at NCH. When data is accessed from the iPads and stored on a secure server on the NCH network, the data will be deleted from the iPads.

# Procedures Involved*

- 1. Describe and explain the study design.


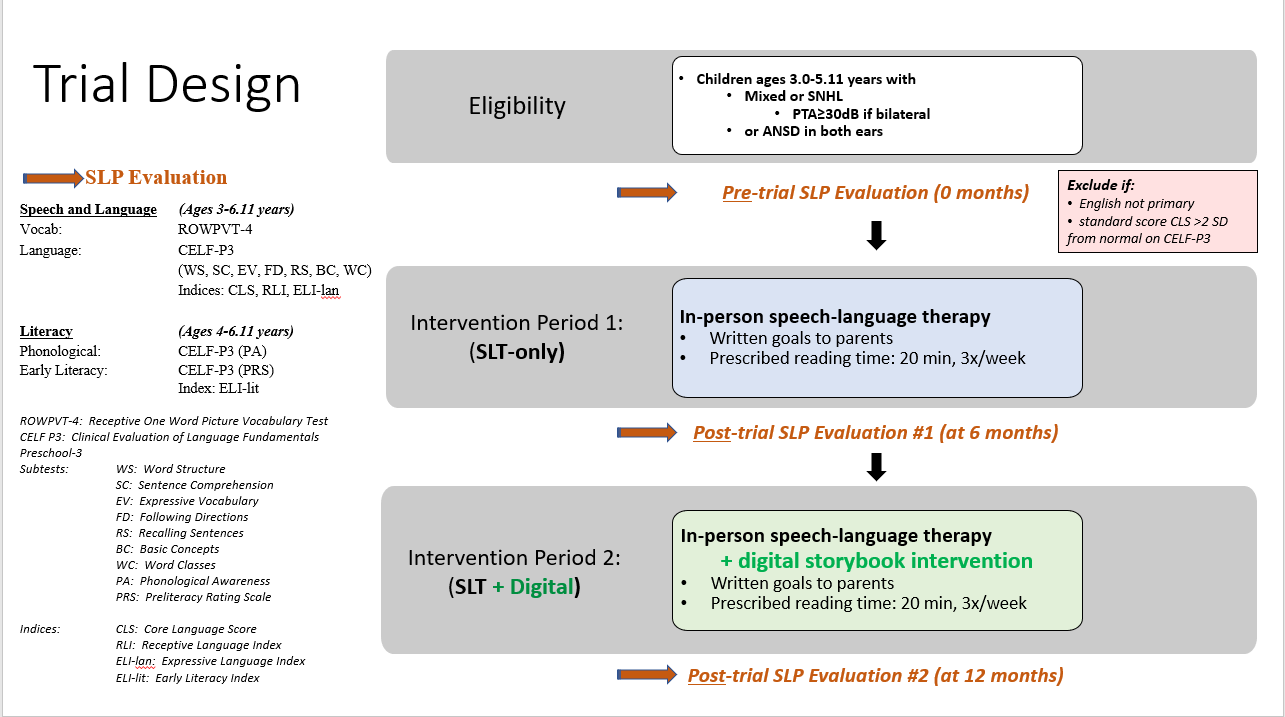
This project will use a prospective, two-period design, summarized in **Figure 2.** Treatment and outcome measures are unblinded. There is no washout period – due to clinical and insurance standards and validated outcome measures designed to be performed every 6 months. This would impose an unmanageable timeline. To eliminate any potential carryover effects that may occur from SLT+Digital to SLT-only (but are not expected to occur in reverse), we will enroll all children and their parent/caregiver **sequentially** into SLT-only period first, and then intervention period with SLT+Digital. After informed consent is obtained, and completion of the intake and review of inclusion and exclusionary criteria, the child and their parent/caregiver will be sequentially enrolled in: 1) Speech-language therapy alone (SLT-only), **and then** (2) Speech-language therapy with digital storybook intervention (SLT+Digital). The recruitment plan will aim for N=50 subjects.

**Figure 2**

- 1. Provide a description of all research procedures being performed and when they are performed, including procedures being performed to monitor subjects for safety or minimize risks.

Our study will conform to the protocol as approved by our Institutional Review Board (IRB). Upon approval by the NCH IRB, recruitment efforts will begin.

Study materials (e.g., study packets) for all participants will be offered in paper and virtual formats.

**Child and Parent/Caregiver Participants:**

Study staff, audiologists, SLPs, nurses, and physicians, will work together and with the EDW to identify eligible participants based on review of clinic schedules and chart reviews. Recruitment will be done by study coordinators (NCH Behavioral Trials Office, or BTO).

*Participation:*

- After informed consent is obtained, and completion of the intake and review of inclusion and exclusionary criteria, families will be given a **Study Packet** **(Parent/Caregiver)** at the beginning of each intervention period**.** Specific materials will depend on the intervention period (pre-SLT or post-SLT) and may include study information, survey information, clinical recommendations, and training documents (**Study Checklist – Parent/Caregiver**, **HMR Visual Timeline**, **Study** **Review Handout - Caregiver**, **Pre-Trial Parent/Caregiver Survey**, **copy of** **Informed Consent** **Form – Parent/Caregiver** with study/IRB contact information, **American Academy of Pediatrics brochure - “Beyond Screen Time – A Parent’s Guide to Media Use”** sheet, and the **Prescribed Reading Flyer**. The **Hear** **Me Read – User Manual** and link to the **Hear Me Read Training video** will be provided in this packet as well.) Study coordinator will assist with HMR training as needed.
- The child will participate in a **Pre-trial SLP Evaluation** consisting of a battery of clinical measures as part of standard clinical care (ROWPVT-4, CELF-P3) for D/HH children. These are behavioral tests, administered by a SLP to assess a child’s language and communication skills**.** This evaluation will be performed by a treating SLP (SLP_T_) as part of standard care, at a standard therapy visit.
- After **Pre-trial SLP Evaluation**, each child will be enrolled into **SLT-only intervention for 6 months (Intervention Period 1)**, the clinically standard test-retest interval for serial behavioral speech-language and literacy assessments in D/HH children. In-person therapy frequency is determined clinically by SLP_T_ and may range from weekly to every 6-months, but frequency is expected to be the same time period for Intervention Period 1 and Intervention Period 2. All of the sessions in this study will be done as part of standard of care visits. There are no additional data collection in-person research visits, unless necessary to collect survey data or requested by the participating caregiver. The SLT-only intervention period is current standard of clinical care with in-person speech-language therapy with SLP_T_, and written goals provided to parents/caregivers based off of individualized pre-trial evaluation. Standardized reading time will be recommended, and prescribed at 20 min, 3x/week, to try to minimize differences between the treatment groups.
- The parent/caregiver will fill out the brief **Reading Questionnaire** at least monthly during the study, to assess parent/caregiver-reported reading habits. The SLP_T_ may collect this information at each speech therapy session.
- After 6 months of therapy, the clinical standard is for re-evaluation (**Post-SLT-only SLP Evaluation**) with the clinical measures (such as ROWPVT-4, CELF-P3). SLP_T_ will complete re-evaluations at the end of **Intervention Period 1**. A study SLP (SLP_S_), who is not involved in providing therapy as a SLP_T_, will validate the scores provided by the SLP_T_. Scores on the ROWPVT-4 and the CELF-P3 will be collected in the **Therapist - Data collection Form** , built in REDCap. SLP_T_ will record evaluations on a NCHa device connected to NCH’s secured network. Once recorded, video files will be stored on a secured encrypted NCH drive, accessible only to study team members and the SLP_T_. Once the video is uploaded to the NCH server, it will be deleted from the device.
- Parent/caregiver survey (**Post-SLT-only-Parent/Caregiver**) on the therapy experience will be filled out after the intervention period.
- Children are then continued in the next phase of the study (**Intervention Period 2)** with the **SLT+Digital intervention** **for 6 months**. In the SLT+Digital phase, the child will receive in-person therapy in identical fashion as the SLT-only intervention period with a SLP_T,_ and also with the same prescribed reading time. However, the SLP_T_ will now **additionally** assign storybooks through HMR. Difficulties using HMR will be directed to Research Information Solutions and Innovation (RISI) at The Research Institute at NCH who is budgeted for support, along with study coordinator. Contact information will be in the **Hear Me Read Training document.**
- At the conclusion of **Intervention Period 2 (Post-SLT+Digital SLP Evaluation)**, a final assessment battery is completed by the SLP_T_ as part of standard clinical care. Scores on the ROWPVT-4 and the CELF-P3 will be collected in the **Therapist - Data collection Form**, built in REDCap.
- SLP_T_ will record evaluations on an NCH device connected to NCH’s secured network. Once recorded, video files will be stored on a secured encrypted NCH drive, accessible only to study team members and the SLP_T_. Once the video is uploaded to the NCH server, it will be deleted from the device.
- To minimize bias, SLP_S_ will be unaware if the re-evaluation is Post-SLT-only or Post-SLT+Digital, whenever possible.
- As before, a survey (**Post-SLT+Digital -Parent/Caregiver**) on the therapy experience will be filled out by the parent/caregiver after the intervention period.
- Parents/caregivers will additionally fill out **Hear Me Read Feedback-Parent/Caregiver** and questionnaires to provide user feedback on the digital platform.

**Treating SLP Participants:**

*Participation:*

- The SLP_T_ will be provided Study Packet (Treating Therapist), which includes study information, survey information, and training documents(**Study Checklist – Treating Therapist, Study Review Handout - Treating Therapist , Hear Me Read Treating Therapist Manual**, **Reading Questionnaire).** Additionally, the research team will provide SLP_T_ with a study iPad on which they will record speech evaluations. Study coordinator will assist with HMR training as needed.
- They will complete treating SLP surveys (**Post-SLT-only-Treating Therapist**) on the therapy experience after the SLT-only intervention period. One set of surveys will be completed for each participating child.
- They will complete treating therapist survey (**Post-SLT+Digital -Treating Therapist**) on the therapy experience after the SLT+Digital intervention period. One set of surveys will be completed for each participating child.
- They will provide user feedback on the digital platform (**Hear Me Read Feedback-Treating Therapist)**. One set of surveys will be completed for each participating child.

**SMS Messaging:**

This study will use Twilio, a third-party platform that is integrated with REDCap, to:

- Send text messages with survey links to a REDCap form: Twilio will send a text message to the participant. The message will contain a link to a REDCap form. The text message will not include PHI or PII. The text message will provide a link to the secure REDCap platform. The participant will click on the link and be directed to a secure REDCap platform to complete the survey.
- Informational texts to study participants.
  1. Describe Procedures performed to lessen the probability or magnitude of risks.
- Study staff will provide instruction on Hear Me Read and troubleshoot, so therapy time is minimally affected.
- Young children are more susceptible to harms from screen time; in light of American Academy of Pediatrics recommendations, we are not enrolling children under age 2 years of age
- At time of child enrollment, study personnel will say the following safety statement during the consent process:
  - **Screen Time Caution script**: “The amount of child screen time spent in performing study tasks are designed to be in accordance with American Academy of Pediatrics limits on screen time. Please read these information sheets regarding AAP guidelines on safe screen time use and creating a family Media Use Plan.”
- Study packet will include printed screen safety information:
  - American Academy of Pediatrics brochure - **“Beyond Screen Time – A Parent’s Guide to Media Use”**
    - Description from AAP website: Parents/caregivers look to their pediatricians and other professionals when it comes to guidance on kids and screen time. This brochure describes how television and other screens, like computers, smart phones, and tablets, can affect children. The brochure contains age-specific guidelines from infants to teens, based on AAP policy. It includes the advantages and risks for children in this digital age and helps parents/caregivers make using these devices safe and fun for the whole family.

6.4 The source records that will be used to collect data about subjects. (Attach all surveys, scripts, and data collection forms.)

**Study documents below.**

**Data Source Forms (electronic in REDCap, input from iPad or abstracted from EPIC):**

- Therapist - Data Collection Form (All part of standard clinical care):
  - Pre-Trial SLP Evaluation data (ROWPVT-4, CELF-P3)
  - Post-SLT-only SLP Evaluation data (ROWPVT-4, CELF-P3)
  - Post-SLT+Digital SLP Evaluation data (ROWPVT-4, CELF-P3)
- Participating parent/caregiver and treating therapist surveys:
  - Pre-Trial Parent/Caregiver Survey
  - Post-SLT-only – Parent/Caregiver
  - Post-SLT+Digital – Parent/Caregiver
  - Hear Me Read Feedback – Parent/Caregiver
  - Post-SLT-only – Treating Therapist
  - Post-SLT+Digital – Treating Therapist
  - Hear Me Read Feedback – Treating Therapist
  - Reading Questionnaires (Parent/Caregiver)

**Study Packet (Parent/Caregiver) contents:**

- - - - Study Checklist – Parent/Caregiver
      - Study Review Handout – Caregiver
      - Pre-Trial Parent/Caregiver Survey
      - Copy of Informed Consent Form – Parent/Caregiver with study/IRB contact information
      - American Academy of Pediatrics brochure - “Beyond Screen Time – A Parent’s Guide to Media Use”
      - Hear Me Read – User Manual
      - Reading Questionnaires

**Study Packet contents (Treating Therapist):**

- - - - - Study Checklist – Treating Therapist
        - Study Review Handout – Treating Therapist
        - Hear Me Read – Therapist Manual
        - Reading Questionnaires (to ask of parents at each speech therapy visit)

**Recruitment and Incentives documents:**

- Recruitment Script – Parent/Caregiver
- Recruitment Script – Treating Therapist
- Hear Me Read 2021 Recruitment Email/Letter
- Hear Me Read 2021 Recruitment Flyer
- Survey – Email/Text Intro and Link text
- Thank You Letter

**Hear Me Read Training documents:**

- Hear Me Read – Treating Therapist Manual
- Hear Me Read – Parent/Caregiver User Manual

**Patient Medical and Demographic Information:** Obtained from the electronic medical record at NCH (see Section 27)

- - Records from Audiology, Speech-Language Therapy, ENT
  - General Demographics and PHI (see Section 27)
  - Audiologic measures
  - Speech perception
  - Degree of hearing loss
- Mode of communication (oral, total communication, manual/sign language)
- Etiology of hearing loss
- Technology (Hearing aid, cochlear implant, none) and dates of service
  - Maternal level education

6.5 What data will be collected during the study and how that data will be obtained.

Child participant data to be collected includes:

1. **Primary Outcomes:**

- Behavioral measures include age-appropriate behavioral surveys administered by a SLP to assess a child’s language and communication skills, conducted as part of standard clinical care. These consist of verbal or pointing tasks in response to verbal or picture prompts and questions. These are documented in **Therapist - Data collection Form** containing data **Pre-Trial SLP Evaluation, Post- SLT only SLP Evaluation, and Post-SLT+Digital SLP Evaluation** consisting of **(see Figure 1):**
  - Raw Score, Standard Score, Percentile Rank, Age Equivalent, and Growth Scale Values where applicable for the following tests.
    - **Speech and Language Outcomes**
      - Vocabulary
      - [Receptive One Word Picture Vocabulary Test (ROWPVT-4)](https://www.pearsonassessments.com/store/usassessments/en/Store/Professional-Assessments/Speech-%26-Language/Receptive-and-Expressive-One-Word-Picture-Vocabulary-Tests-%7C-Fourth-Edition/p/100000338.html#:~:text=The%20ROWPVT%2D4%20tests%20an,when%20presented%20with%20color%20illustrations.) (ages 2.0-70+), 10-15 min
      - Language
        - [Clinical Evaluation of Language Fundamentals Preschool-3 (CELF P3)](https://www.pearsonassessments.com/store/usassessments/en/Store/Professional-Assessments/Developmental-Early-Childhood/Clinical-Evaluation-of-Language-Fundamentals-Preschool-3/p/100002031.html?tab=product-details) (ages 3.0-6.11), 15-20 min

Subtests:

Word Structure (WS)

Sentence Comprehension (SC)

Expressive Vocabulary (EV)

Following Directions (FD)

Recalling Sentences (RS)

Basic Concepts (BC)

Word Classes (WC)

Indices (composite scores of selected subtests):

CLS (Core Language Score) (Ages 3-6)

Receptive Language Index (RLI) (Ages 3-4)

Receptive Language Index (RLI) (Ages 5-6)

Expressive Language Index (ELI-lan) (Ages 3-6)

- - - **Literacy Outcomes**
      - Emerging Literacy
        - [Clinical Evaluation of Language Fundamentals Preschool-3 (CELF P3)](https://www.pearsonassessments.com/store/usassessments/en/Store/Professional-Assessments/Developmental-Early-Childhood/Clinical-Evaluation-of-Language-Fundamentals-Preschool-3/p/100002031.html?tab=product-details) (ages **4.0**-6.11), 15-20 min

Subtests:

Pre-literacy Rating Scale (PRS)

Phonological Awareness (PA)

Index (composite scores of selected subtests):

Early Literacy Index (composite) (ELI-lit) (Ages 4-6)

1. **Secondary Outcomes:**
   - Measures of the impact on therapy experience, obtained from:

- App measured (see below In-App Metrics)
- **Pre-Trial Parent/Caregiver Survey**
  - **Post-SLT-only – Parent/Caregiver**
  - **Post-SLT+Digital – Parent/Caregiver**
  - **Post-SLT-only – Treating Therapist**
  - **Post-SLT+Digital – Treating Therapist**
  - **Hear Me Read Feedback-Parent/Caregiver**
  - **Hear Me Read Feedback-Treating Therapist**
  - **Reading Questionnaires**
- Book Engagement/book-reading habits, obtained from:
  - App measured (see below In-App Metrics)
- **Pre-Trial Parent/Caregiver Survey**
  - **Post-SLT-only – Parent/Caregiver**
  - **Post-SLT+Digital – Parent/Caregiver**
  - **Post-SLT-only – Treating Therapist**
  - **Post-SLT+Digital – Treating Therapist**
  - **Reading Questionnaires**
- Design and usability of the Hear Me Read app, obtained from:
  - App measured (see below App-Measured Metrics) form Hear Me Read app
  - **Pre-Trial Parent/Caregiver Survey**
  - **Hear Me Read Feedback-Parent/Caregiver**
  - **Hear Me Read Feedback-Treating Therapist**

1. **Patient Medical and Demographic Information:**

- Obtained from the electronic medical record at NCH (see Section 27)
- Records from Audiology, Speech-Language Therapy, ENT
- General Demographics and PHI (see Section 27)
- Audiologic measures
  - Speech perception
  - Degree of hearing loss
- Mode of communication (oral, total communication, manual/sign language)
- Etiology of hearing loss
- Technology (Hearing aid, cochlear implant, none) and dates of service
  - Maternal level education

1. **App-Measured Metrics, from Hear Me Read App**

- # books available
- Lings done?
- Book richness/enhancements:
  - Amount of audio
  - Amount of video
  - # of parsed words in the book
  - # categories and words in categories
  - Lexile score
  - Highlights – word count
  - Highlights – themes/topics
  - Highlights – fiction/nonfiction
  - Highlights – category type (enhanced, etc)
- Time:
  - App launch
  - App close
  - When being used (time of day)
  - Time spent doing narration
  - #times book opened
  - Time in app overall
  - Time spent in book
- What’s being consumed:
  - % of book read (each time)
  - #times any word is clicked in a book per session
    - Audio
    - Visual
  - Which categories are clicked (how many and what)

6.6 If there are plans for long-term follow-up (once all research related procedures are complete), what data will be collected during this period.

**N/A**

6.7 For Humanitarian Use Device (HUD) uses provide a description of the device, a summary of how you propose to use the device, including a description of any screening procedures, the HUD procedure, and any patient follow-up visits, tests or procedures.

**N/A**

# Data and Specimen Banking*

7.1 If data or specimens will be banked for future use, describe where the specimens will be stored, how long they will be stored, how the specimens will be accessed, and who will have access to the specimens.

We will store identifiable information (including PHI) for future research purposes.

Data from this study, including PHI related to research, will be stored for 6 years after conclusion of the study.  Data will be stored in a secured folder on the NCH network and REDCap servers, accessible to members of the study team. Study staff will access the information after logging into the NCH secure network.

7.2 List the data to be stored or associated with each specimen.

**N/A** – no specimens to be stored

7.3 Describe the procedures to release data or specimens, including: the process to request a release, approvals required for release, who can obtain data or specimens, and the data to be provided with specimens.

**N/A** – no specimens to be stored

# Sharing of Results with Subjects*

8.1 Describe whether results (study results or individual subject results, such as results of investigational diagnostic tests, genetic tests, or incidental findings) will be shared with subjects or others (e.g., the subject’s primary care physicians) and if so, describe how the results will be shared.

Speech, language, and literacy outcome measures that are collected as part of routine clinical care will be reported to the family, as part of standard care by the therapists. Typical clinical documentation and correspondence with referring and treating providers will be maintained.

# Study Timelines*

- 1. Describe: The duration of an individual subject’s participation in the study.

Each child is anticipated to participate in 2 separate 6-month intervention periods (Intervention Period #1/SLT-only and Intervention Period #2/SLT+Digital), for a total of 12 months. See **Figure 2**. SLP visits may be either evaluations or therapy sessions and are part of standard care. Evaluations at the start of therapy and in 6 month intervals may take 2-2.5 hours each (such as the **Pre-Trial SLP Evaluation, Post-SLT SLP Evaluation, and Post-SLT + Digital SLP Evaluation)**. In-person therapy frequency is determined by SLP_T_ and may range from monthly to weekly. Duration of therapy sessions are typically 1 hour. All of the sessions in this study will be done as part of standard of care visits. There are no additional research visits, unless necessary to collect survey data or requested by the participating caregiver.

- 1. Describe: The duration anticipated to enroll all study subjects.

Feasibility analysis of local subjects (Section 12) indicates 411 D/HH children were seen in 2019 in the age group we are examining. Of these, 141 children had a speech and language evaluation billed in that year. Our power analysis motivates us to recruit a total of 50 patients. It we account for eligibility, choice of participation, and patient volume, we anticipate approximately **9 months-1 year** to recruit fully.

- 1. Describe: The estimated date for the investigators to complete this study (complete primary analyses)

With rolling recruitment and intervention, expected recruitment, and 1 year treatment period, we anticipate approximately **2-2.5 years from start of study** to completion of primary analysis.

# Inclusion and Exclusion Criteria*

Children seen in the Hearing Program at Nationwide Children’s Hospital who are deaf/hard of hearing are eligible. This includes Main Campus and off-site locations, in the departments that make up the multidisciplinary Hearing Program (Pediatric Otolaryngology, Audiology and Speech-Language Therapy). Additionally, their parent/caregiver and treating SLP will be invited to participate.

**Child Eligibility:**

- ***Inclusion:***
  - Children ages 3.0-5.11 years at time of initial assessment and
    - Auditory neuropathy in both ears, or
    - Bilateral Sensorineural or mixed hearing loss at least (PTA≥30dB)
- ***Exclusion:***
  - English not primary language (app is English only at present)
  - standard score CLS >2 SD from normal on CELF-P3

**Parent/Caregiver Eligibility:**

- ***Inclusion:***
  - Parent/caregiver of eligible child (must be child’s legal guardian)
- ***Exclusion:***
  - None

**Treating SLP Eligibility:**

- ***Inclusion:***
  - Treating therapist/SLP for participating child
- ***Exclusion:***
  - None

# Vulnerable Populations*

11.1 If the research involves individuals who are vulnerable to coercion or undue influence, describe additional safeguards included to protect their rights and welfare.

This research study does NOT involve pregnant women, neonates, prisoners, or cognitively impaired adults.

This research involves no greater than minimal risk for children or adults.

PHI will only be shared with the groups listed below:

- PI and Study Staff
- The Nationwide Children’s Hospital Institutional Review Board (the committee that reviews all human subject research)
- Nationwide Children’s Hospital internal auditors
- Participant’s insurance company

PHI will be protected in the following ways:

- Research records will be stored in a locked cabinet in a secure location
- Research records will be stored in a password-protected computer file
- The list linking the assigned code number to the individual subject will be maintained separately from the other research data
- Only certified research personnel will be given access to identifiable participant information

# Local Number of Subjects

12.1 Indicate the total number of subjects to be accrued locally.

A **power analysis** was performed, based off of Raw Scores with the following rationale: A repeated measures design will evaluate the effect of the intervention within person. In this case, the primary goal of the study is to compare the change across time during SLT therapy alone to the change across time with SLT + Digital. A sample size of 43 achieves 90% power to detect a mean of paired differences of 0.75 with an estimated standard deviation of 1.0 and with a significance level of 0.001 using a two-sided paired t-test.   We plan to enroll 50 children at NCH, as well as a parent/caregiver for each child and the child’s treating SLP.

12.2 If applicable, distinguish between the number of subjects who are expected to be enrolled and screened, and the number of subjects needed to complete the research procedures (i.e., numbers of subjects excluding screen failures.)

We expect approximately 411 unique D/HH children in the age range to be seen by our Hearing Program in 1 year, based on 2019 data. Of these, 141 completed a speech and language evaluation in that year and 270 did not. With prospective attention to scheduling and these volumes, we anticipate screening approximately 250-300 patients to achieve 50 that would enroll.

# Recruitment Methods

- 1. Describe when, where, and how potential participants will be recruited.

Participation is completely voluntary; refusal to participate will involve no penalty or loss of benefits to these potential subjects. No attempts at coercion will be made.

**Child and Parent/Caregiver Participants:**

Upon approval by the NCH IRB, recruitment efforts will begin. Potential patients will be identified by study staff, audiologists, SLPs, nurses, and physicians in the ENT department at NCH, along with data requests obtained from the EDW at NCH. Study eligibility and exclusionary criteria will be made known to those involved in identifying eligible participants individuals in advance.

Written documentation of informed consent will be obtained and documented from parents/caregivers or legal guardians of each prospective participant who meet inclusion and exclusion criteria who agree to participate.

This study will implement a multi-pronged (3 strategies described below) recruitment approach for child and parent/caregiver participants.

Eligibility will be assessed in EPIC based on child’s date of birth, diagnosis, primary language, treatment, treatment dates, treatment location, providing clinicians, and speech therapy assessment scores. After identification via EPIC and/or EDW, clinicians will be notified about potentially eligible participants on their schedule. During the appointment, clinicians will inquire if the family agrees to be contacted by the study coordinator for more information about the Hear Me Read study. If family agrees, study staff will be notified and will meet with/contact the family to review the study information and recruit the family using the **Recruitment Script** as a guide. This may be done while the family is on-site or via phone or email after their visit. If research staff is unable to meet family at the clinic to review study information and recruit after family verbally agrees to be contacted, research staff will contact family via phone call and/or email. If the family wishes to participate, eligibility is assessed and enrollment proceeds if confirmed.

In addition to identifying eligible children in advance of their clinical appointment, recruitment will also be done by posted and digital flyers. A paper recruitment flyer (**Hear Me Read 2021 Recruitment Flyer)** will be placed in public areas of targeted NCH facilities (such as clinical therapies, ENT, Audiology) at Main and Close To Home locations so that families may know about and volunteer to participate. Digital version of **Hear Me Read 2021 Recruitment Flyer** will be sent through NCH distribution channels through the Research Institute and Marketing. No incentive is provided to post. Study coordinator information will be on the flyers and interested families will be instructed to contact the study coordinator.

The **Hear Me Read 2021 Recruitment Email/Letter** will be sent to established patients in the NCH Hearing Program. Emails may be sent via myChart, in line with hospital policies for this recruitment method or via direct email or mail contact. Study coordinator information will be included in the email/letter and interested families will be instructed to contact the study coordinator. Study staff will contact (e.g., call, text, email) caregiver approximately one to two weeks after the initial email or letter has been sent if study staff does not receive a response. The email/letter offers an option to opt-out if caregiver wishes to receive no further information about the study.

***Treating SLP Participants:***

BTO study staff will reach out to Speech Language Pathologists who treat eligible children. This includes SLPs in the departments that make up the multidisciplinary Hearing Program (Pediatric Otolaryngology, Audiology and Speech-Language Therapy) and those external to NCH. Study staff will contact SLPs via phone and/or email to provide information about the HMR study.

Consent will be obtained consistent with the Waiver of Written Documentation regulations as outlined in Section 22 of this protocol. Participating Speech Language Pathologists will receive a SLP packet for each enrolled child participant.

13.2 Describe the source of subjects.

The children in this study will consist of D/HH patients in the Hearing Program at NCH, seen through the ENT, audiology, and Speech-language Therapy departments. Their parent/caregiver, as well as their treating SLP will also be invited to participate.

13.3 Describe the methods that will be used to identify potential subjects.

See above, section 13.1

13.4 Describe materials that will be used to recruit subjects. (Attach copies of these documents with the application. For advertisements, attach the final copy of printed advertisements. When advertisements are taped for broadcast, attach the final audio/video tape. You may submit the wording of the advertisement prior to taping to preclude re-taping because of inappropriate wording, provided the IRB reviews the final audio/video tape.)

- **Hear Me Read 2021 Recruitment Flyer**
- **Hear Me Read 2021 Recruitment Email/Letter**

13.5 Describe the amount and timing of any payments to subjects.

Incentive ($10/survey) will be offered to children’s’ parents/caregivers and treating speech-language pathologists, and provided upon completion of surveys. We will use GreenPhire Clincards through NCH as incentives for completion of surveys as follows:

- - Treating Therapists:
    - Post-intervention #1 (SLT) - Treating Therapist at 6 months
    - Post-intervention #2 (SLT+Digital) – Treating Therapist at 12 months
  - Parents/Caregivers
    - Pre-trial parent/caregiver survey – Parent/Caregiver at enrollment
    - Post-intervention #1(SLT) - Parent/Caregiver at 6 months
    - Post-intervention #2 (SLT+Digital) – Parent/Caregiver at 12 months

Participants can choose to not provide this, without penalty. Surveys will be electronic, done via REDCap.

# Withdrawal of Subjects*

14. 1 Describe anticipated circumstances under which subjects will be withdrawn from the research without their consent.

Participation is voluntary, and can be terminated at any time by participants. This study has minimal risk to patients, so unexpected withdrawal due to safety reasons from the study design is not expected to occur.

14.2 Describe any procedures for orderly termination.

The request for termination will be managed and documented by the study coordinator. Study personnel, study and treating therapists will be notified.

14.3 Describe procedures that will be followed when subjects withdraw from the research, including partial withdrawal from procedures with continued data collection.

The study coordinator will be made aware of the withdrawal and coordinate this. The coordinator will coordinate the return of study equipment by the family. We will retain study data related to the subject collected prior to the subject's withdrawal from the study. Study-related events scheduled after the withdrawal date will be cancelled and no further data collected.

# Risks to Subjects*

15.1 List the reasonably foreseeable risks, discomforts, hazards, or inconveniences to the subjects related the subjects’ participation in the research. Include as may be useful for the IRB’s consideration, a description of the probability, magnitude, duration, and reversibility of the risks. Consider physical, psychological, social, legal, and economic risks.

Participants can choose to not participate, and this will have no consequences to clinical care or employment.

The duration of time spent using screens/digital devices is important to consider. The prescribed reading time using the HMR app is not in excess of recommendations made by the American Academy of Pediatrics, and we are not enrolling children aged 2 or younger (who are at greatest risk). To encourage healthy overall screen use, our study packet will include the following screen safety information:

- - American Academy of Pediatrics information: AAP - Tips for Parents in the Digital Age and Media Use Plan
    - [Children and Media - Tips for Parents​](https://www.healthychildren.org/English/family-life/Media/Pages/Tips-for-Parents-Digital-Age.aspx):
      - <https://www.healthychildren.org/English/family-life/Media/Pages/Tips-for-Parents-Digital-Age.aspx>
    - Making a Media Use Plan:
      - [www.HealthyChildren.org/MediaUsePlan](http://www.healthychildren.org/MediaUsePlan)

Economic risks are not expected to be excessive, given that interventions will take place during standard clinical care visits. No additional parking or transportation needs are expected. Time spent learning and troubleshooting the software could occur. We will provide contact information to the Research Information Solutions and Innovation (RISI) at The Research Institute at Nationwide Children's Hospital, who is budgeted to provide ongoing support for this purpose.

Although we will take every precaution, there is a small chance of loss of confidential study information. The study staff, and Nationwide Children’s Hospital technology teams, have taken precautions to ensure all the information, including videos, collected on the iPad are stored and transferred securely.

15.2 If applicable, indicate which procedures may have risks to the subjects that are currently unforeseeable.

**N/A** – no more than Minimal Risk to subjects

15.3 If applicable, indicate which procedures may have risks to an embryo or fetus should the subject be or become pregnant.

**N/A**

15.4 If applicable, describe risks to others who are not subjects.

**N/A**

# Potential Benefits to Subjects*

16.1 Describe the potential benefits that individual subjects may experience from taking part in the research. Include as may be useful for the IRB’s consideration, the probability, magnitude, and duration of the potential benefits.

At this time, little is known about digital reading interventions in young D/HH children. Potentially, structured prospective follow up with children in this study could decrease the loss to follow up and improve compliance with speech-language therapy that is prescribed. Increased reading and book engagement may occur, and this generally provides educational and developmental benefits in all children, including those that are D/HH.

16.2 Indicate if there is no direct benefit. Do not include benefits to society or others.

There may be no direct benefit.

# Data Management* and Confidentiality

17.1 Describe the data analysis plan, including any statistical procedures or power analysis.

Raw Score, Standard Score, Percentile Rank, Age Equivalent, and Growth Scale Values for the assessment tests will be measured. Power analysis was based off of Raw Score. A 2-by-2 repeated measures design consists of two groups of subjects, each measured at two time points. In this case, the primary goal of the study is to compare the change across time in group 1 to the change across time in group 2. Sample sizes of 13 in group 1 and 13 in group 2 achieve 92% power to detect a difference in mean changes of 2.0 with a standard deviation of 1.0 at the first time point, a standard deviation of 1.0 at the second time point, and a correlation between measurement pairs of 0.300. The significance level (alpha) is 0.010 using a two-sided, two-sample t-test. To detect a mean 1.5 point difference (all else being equal), we would need **21 patients in each group**. To detect a mean 1 point difference (all else being equal), we calculate we would need 44 patients in each arm.

We plan to analyze Intervention Period 1 (SLT) and Intervention Period 2 (SLT+Digital) together using mixed-effects models to account for differences between interventions 1 and 2. For the primary outcomes, evaluate baseline assessments and compare values between groups using Pearson chi-square tests, t-tests, and Wilcoxon-Mann-Whitney tests. If any imbalance in patient characteristics (e.g. age, frequency of therapy) is detected between groups, the imbalanced characteristics will be adjusted for in multivariable mixed-effects linear regression analyses.

All statistical analyses will be conducted using SAS Enterprise Guide, version 7.15 (SAS Institute Inc). Outcomes are reported for the intention-to-treat population and the also reported for the per protocol population. A 2-sided P < .05 was considered statistically significant.

17.2 Describe the steps that will be taken to secure the data (e.g., training, authorization of access, password protection, encryption, physical controls, certificates of confidentiality, and separation of identifiers and data) during storage, use, and transmission.

Privacy and security will be maintained by minimizing the amount of identifiable data as much as possible. All demographic, clinical, and outcome data will be recorded by local study staff in the study’s central REDCap database maintained at NCH. REDCap is a network software suite that provides data storage, encryption, and password protection for health information, to which only study staff will have access. Any collected paper information will be entered and compiled in an Excel or REDCap database (or password protected computer files). Paper copies will be stored in locked study cabinets until entry, and then shredded in appropriate, in secure NCH bins after electronic capture. Files and video/audio recorded materials will be maintained on computers located at NCH that are maintained on a secure network. Only approved research personnel will be given access to identifiable participant information. All necessary identifiers will be used only to identify the relevant source documents. At the conclusion of data collection, a unique identifier will be assigned to each participant and the identifiers will be deleted from the primary dataset and stored in a separate list. A list linking the assigned code number to the individual subject will be maintained separately from the other research data.

NCH study iPads will be provided to intervention study patients directly, at first therapy visit when HMR intervention is initiated. Hear Me Read will be used as prescribed by a treating speech-language therapist. The iPads provided to study patients that have Hear Me Read software will be NCH study iPads, managed and supported by Research Information Solutions and Innovation (RISI) at The Research Institute at Nationwide Children's Hospital. Serial numbers of the iPads will be documented and stored. These iPads will be password protected, requiring input password before access to any apps. Passwords will be provided only to therapists, study staff, investigators, and to the study family. Upon completion of the study, investigators will store the iPads in a locked drawer, in a locked office at NCH. When data is accessed from the iPads and stored on a secure server on the NCH network, the data will be deleted from the iPads. In-app metrics will be taken from the iPads upon return of the devices, minimizing transfers of information regarding use of the app to a single event. This transfer would be done on secure NCH servers and networks by RISI staff.

The data set will be maintained for 6 years after the study closure.

Data, including audio and video data, will not be stored for future research, unless written permission doing so is voluntarily granted during the informed consent process.

17.3 Describe any procedures that will be used for quality control of collected data.

A trained speech-language pathologist will validate the post-intervention assessment battery of tests (vocabulary, speech, language, literacy outcome measures) for all of the study patients, ensuring consistent data collection. This SLP_s_ is not involved in the treatment of patients and is trained and familiar with the outcome measures since they are used as part of clinical practice.

A study coordinator will ensure that organization and execution of the study follows the protocol, and the surveys/questionnaires are administered.

17.4 Describe how data or specimens will be handled study-wide:

**N/A** – this is a single site study

# Provisions to Monitor the Data to Ensure the Safety of Subjects*

This section is required when research involves more than Minimal Risk to subjects.

N/A – no more than Minimal Risk to subjects

# Provisions to Protect the Privacy Interests of Subjects

19.1 Describe the steps that will be taken to protect subjects’ privacy interests. “Privacy interest” refers to a person’s desire to place limits on whom they interact or whom they provide personal information.

Privacy and security will be maintained by minimizing the amount of identifiable data as much as possible. Only certified research personnel will be given access to identifiable participant information. All necessary identifiers will be used only to identify the relevant source documents. At the conclusion of data collection, a unique identifier will be assigned to each participant and the identifiers will be deleted from the primary dataset and stored in a separate list. A list linking the assigned code number to the individual subject will be maintained separately from the other research data.

19.2 Describe what steps you will take to make the subjects feel at ease with the research situation in terms of the questions being asked and the procedures being performed. “At ease” does not refer to physical discomfort, but the sense of intrusiveness a subject might experience in response to questions, examinations, and procedures.

No attempt to contact participants will be made beyond the contact in the study period.

Information will not be shared with anyone other than study personnel, or used for any other purpose other than the IRB approved purposes.

This information about privacy is provided to the families as part of the study and consent form.

Questionnaires filled by the participants are voluntary.

The participants will only potentially interact with a PI directly during the recruitment and informed consent process.

The PI may be familiar with some families and SLPs from their time in the clinic. Recruitment and consent activities will be conducted by trained Clinical Research Coordinators.

19.3 Indicate how the research team is permitted to access any sources of information about the subjects.

Study staff will use electronic medical records, a NCH secured shared drive, and REDCap to access confidential patient information. These systems will require NCH login. Access to these systems will be done only on NCH computers, and by study personnel.

Only trained and approved research personnel will be given access to identifiable participant information, including audio and visual recordings of participants. After completion of data collection and input into the limited data set, their charts will not be accessed for further data collection.

# Compensation for Research-Related Injury

**N/A** – no more than Minimal Risk to subjects

# Economic Burden to Subjects

21.1 Describe any costs that subjects may be responsible for because of participation in the research.

Participants are not expected to incur additional costs as a results of this study.

Economic risks are not expected to be excessive, given that interventions will take place during standard clinical care visits. No additional parking or transportation needs are expected.

Time spent learning and troubleshooting the software could occur. We will provide contact information to the Research Information Solutions and Innovation (RISI) at The Research Institute at Nationwide Children's Hospital, who is budgeted to provide ongoing support for this purpose.

Families will be provided study iPads and chargers.

No Wifi or internet is needed for use of the HMR app.

# Consent Process

22.1 Indicate whether you will you be obtaining consent, and if so describe:

**Child and Parent/Caregiver Participants:**

We will follow SOP: Written Documentation of Consent (HRP-091).

Potential subjects will be approached at a NCH site or via phone or email after identification and referral to study staff. Study personnel (not the PI) will discuss the study and review the consent form (in person, virtually, or over the phone) with the legal guardian or legally authorized representative. Please refer to the attached document: **Informed Consent** **Form.**

The written form and study information will inform potential participants about the details of the study and written in plain language at a level that reflects the reading ability of potential participants. The informed consent document will be reviewed and signed electronically, via REDCap. A copy will be emailed to the participant. The informed consent process may take place at NCH, virtually through a NCH-approved videoconferencing platform or via phone. A copy of **Informed Consent** **Form** will be provided to families as part of their study packet. Information about the study and researchers, including contact information, will be provided in case the family wishes to review documents on their own time and pace and notify the study staff of participation.

This study does not adversely affect the rights and welfare of subjects, and does not involve more than minimal risk.

If at any of the therapy visits, parents indicate they wish to withdraw from the study or consent, they may do so without retaliation.

No assent will be obtained from the children in this study, as all children will be under age 7 during the study period.

**Treating SLP Participants:**

The study team will contact treating SLPs who work with eligible children and invite them to participate in the study. Beyond standard of care, treating SLPs will be asked to complete 2 electronic surveys via REDCap for each child participant with whom they work.

We request a waiver of written documentation of informed consent. We qualify because:

- The research is not FDA-regulated.
- The research does not involve non-viable neonates.
- Participation involves completing online surveys the research involves no more than minimal risk.
- The research could not be carried out without using identifiable private information because we will need to obtain contact information to invite participants to participate.
- The waiver will not adversely affect the rights and welfare of participants because study staff will discuss the purpose of the study, how we will protect information, that the study is voluntary and will not impact their care or employment at NCH, and the participant’s right to withdraw at any time before sending the surveys to the participant. Treating Speech and Language Therapists will be provided with study information in oral (scripted) and written formats.
- Whenever appropriate, the subjects will be provided with additional pertinent information after participation. – NA, participants will complete a series of questionnaires and responses will be analyzed for the cohort, rather than individual participants. Therefore, there is no information to share with participants after participation.
- Waiver of consent for the storage, maintenance, or secondary research use of the identifiable private information or identifiable biospecimens cannot be granted for those who refused to provide broad consent. – NA, this study does not involve broad consent.

# Process to Document Consent in Writing

We will be following “SOP: Written Documentation of Consent (HRP-091)” for child and parent/caregiver participants.

Our research presents no more than minimal risk of harm to subjects and involves no procedures for which written documentation of consent is normally required outside of the research context.

See consent document **Informed Consent** **Form**.

# Setting

- 1. Describe the sites or locations where your research team will conduct the research.

Identification and recruitment of subjects will occur via EPIC and in the clinical areas of Audiology, Speech-language Therapy, and Otolaryngology at Nationwide Children’s Hospital. The conduct of the study will occur mainly at NCH in areas such as the outpatient clinical areas of Clinical Therapies at Nationwide Children’s Hospital and JWest Clinical Research Space. Researchers from these departments and the Research Institute at Nationwide Children’s Hospital will be involved in carrying out this study.

#

# Resources Available

- 1. Describe the resources available to conduct the research: For example, as appropriate:

The Hearing and Implant Program at Nationwide Children’s Hospital sees a large volume of D/HH children of all ages in ENT, Audiology and Speech-language Therapy. This is approximately 2500-3000 children from Ohio and surrounding states. The clinicians and researchers have the appropriate knowledge base and dedicated research time to complete this study. Our clinical office spaces support high volumes of clinical throughput.

We expect approximately 411 unique D/HH children in the age range to be seen by our Hearing Program in 1 year, based on 2019 data. Of these, 141 completed a speech and language evaluation in that year and 270 did not. With prospective attention to scheduling and these volumes, we anticipate screening approximately 250-300 patients to achieve 50 that would qualify.

Additionally, we will collaborate with partners in Research Information Solutions and Innovation (RISI) at The Research Institute at Nationwide Children’s Hospital to provide additional expertise and software support. All staff involved in research will be required to review the IRB protocol and all personnel will be approved by the IRB prior to reviewing any identifiable data.

The Behavioral Trials Office (BTO) at the Research Institute at NCH will be budgeted to provide a trained study coordinator, and statistical support. The Clinical Research Services is engaged and can also help.

Additionally, Dr. Prashant Malhotra will act as an expert consultant, available to the study team for pertinent questions about hearing loss and respective diagnoses. Potential subjects will be informed about the former PIs, Dr. Prashant Malhotra, conflict of interest (COI). The informed consent form will include the COI CMP required disclosure on the matter. Dr. Malhotra will not be involved in study-related activities (i.e., screening, recruitment, consent, or other PI-relevant responsibilities). Dr. Prasanth Pattisapu will take over PI responsibilities, and Dr. Tendy Chiang will assist.

# Multi-Site Research*

# ****N/A****

# ****27.0 Protected Health Information Recording****

1. **Indicate which subject identifiers will be recorded for this research.**

Name

Complete Address

Telephone or Fax Number

Social Security Number (do not check if only used for ClinCard)

Dates (treatment dates, birth date, date of death)

Email address , IP address or url

Medical Record Number or other account number

Health Plan Beneficiary Identification Number

Full face photographic images and/or any comparable images (x-rays)

Account Numbers

Certificate/License Numbers

Vehicle Identifiers and Serial Numbers (e.g. VINs, License Plate Numbers)

Device Identifiers and Serial Numbers

Biometric identifiers, including finger and voice prints

Other number, characteristic or code that could be used to identify an individual

- Video and audio recording of standard-of-care clinical assessments

None (Complete De-identification Certification Form)

**2.0  Check the appropriate category and attach the required form* on the Local Site Documents, #3. Other Documents, page of the application.  (Choose one.)**

Patient Authorization will be obtained. (Include the appropriate HIPAA language (see Section 14 of consent template) in the consent form OR attach the HRP-900, HIPAA AUTHORIZATION form.)

Protocol meets the criteria for waiver of authorization. (Attach the HRP-901, WAIVER OF HIPAA AUTHORIZATION REQUEST form.)

Protocol is using de-identified information. (Attach the HRP-902, DE-IDENTIFICATION CERTIFICATION form.) (Checked "None" in 1.0 above)

Protocol involves research on decedents. (Attach the HRP-903, RESEARCH ON DECEDENTS REQUEST form.)

Protocol is using a limited data set and data use agreement. (Contact the Office of Technology Commercialization to initiate a Limited Data Use Agreement.

***Find the HIPAA forms in the IRB Website Library, Templates.**

**Attach the appropriate HIPAA form on the “Local Site Documents, #3. Other Documents”, page of the application.**

1. **How long will identifying information on each participant be maintained?**

**The data set, including PHI related to research, will be maintained for 6 years after the study ends.**

1. **Describe any plans to code identifiable information collected about each participant.**

Privacy and security will be maintained by minimizing the amount of identifiable data as much as possible. Only approved research personnel will be given access to identifiable participant information. All necessary identifiers will be used only to identify the relevant source documents. At the conclusion of data collection, a unique identifier will be assigned to each participant and the identifiers will be deleted from the primary dataset and stored in a separate list. A list linking the assigned code number to the individual subject will be maintained separately from the other research data.

Study staff will have appropriate REDCap permissions.

1. **Check each box that describes steps that will be taken to safeguard the confidentiality of information collected for this research:**

⌧ Research records will be stored in a locked cabinet in a secure location

⌧ Research records will be stored in a password-protected computer file

⌧ The list linking the assigned code number to the individual subject will be maintained separately from the other research data

⌧ Only certified research personnel will be given access to identifiable subject information

**6.0 Describe the provisions included in the protocol to protect the privacy interests of subjects, where "privacy interests" refer to the interest of individuals in being left alone, limiting access to them, and limiting access to their information. (This is not the same provision to maintain the confidentiality of data.)**

Privacy and security will be maintained by minimizing the amount of identifiable data as much as possible. Only certified research personnel will be given access to identifiable participant information. All necessary identifiers will be used only to identify the relevant source documents. At the conclusion of data collection, a unique identifier will be assigned to each participant and the identifiers will be deleted from the primary dataset and stored in a separate list. A list linking the assigned code number to the individual subject will be maintained separately from the other research data.

No attempt to contact families will be made beyond the contact in the study period. Information will not be shared with anyone other than study personnel, or used for any other purpose other than the IRB approved purposes.

This information about privacy is provided to the families as part of the study and consent form.

Questionnaires filled by the family are voluntary.

The participants will only potentially interact with a PI directly during both recruitment and the informed consent process.

The PI may be familiar with some families from their time receiving treatment in the clinic.

**Confidential Health Information**

1. **Please mark all categories that reflect the nature of health information to be accessed and used as part of this research.**

Demographics (age, gender, educational level)

Diagnosis

Laboratory reports

Radiology reports

Discharge summaries

Procedures/Treatments received

Dates related to course of treatment (admission, surgery, discharge)

Billing information

Names of drugs and/or devices used as part of treatment

Location of treatment

Name of treatment provider

Surgical reports

Other information related to course of treatment

None

1. Please discuss why it is necessary to access and review the health information noted in your response above.

Demographic, audiologic, speech, and other medical information that is volunteered by questionnaire or already readily available in the medical records will be collected for use in this study.

The proposed research is a clinical trial in deaf/hard of hearing children with and their interaction with a software platform. The trial will need to be coordinated and executed across multiple Nationwide Children’s Hospital locations. Aspects of medical history (diagnoses, treatments, use of hearing aid, cochlear implant, sign language, language ability, etc) can inform appropriate use of the technology and analysis of results. Response to content could potentially be age- or gender-sensitive, requiring the collection of basic demographic information such as a Date of Birth and gender. Sufficient information will also be needed to ensure fulfillment of inclusion and exclusion criteria.

3.0 Is the health information to be accessed and reviewed the minimal necessary to achieve the goals of this research?  Yes  No

4.0 Will it be necessary to record information of a sensitive nature?  Yes  No

5.0 Do you plan to obtain a federally-issued Certificate of Confidentiality as a means of protecting the confidentiality of the information collected?  Yes  No

# References

***Cited Literature and References***

1. Moeller MP, Tomblin JB, Yoshinaga-Itano C, Connor CM, Jerger S. Current state of knowledge: language and literacy of children with hearing impairment. Ear Hear. 2007 Dec;28(6):740-53. doi: 10.1097/AUD.0b013e318157f07f. PMID: 17982362.
2. Nittrouer S., Caldwell-Tarr A. (2016) Language and Literacy Skills in Children with Cochlear Implants: Past and Present Findings. In: Young N., Iler Kirk K. (eds) Pediatric Cochlear Implantation. Springer, New York, NY. https://doi.org/10.1007/978-1-4939-2788-3_11)
3. Holt J. Classroom Attributes and Achievement Test Scores for Deaf and Hard of Hearing Students. Am Ann Deaf 2012; [doi: 10.1353/aad.2012.0274]
4. Traxler CB. The Stanford Achievement Test, 9th Edition: National Norming and Performance Standards for Deaf and Hard-of-Hearing Students. J Deaf Stud Deaf Educ 2000; [doi: 10.1093/deafed/5.4.337]
5. Wauters LN, Van Bon WHJ, Tellings AEJM. Reading comprehension of Dutch deaf children. Read Writ 2006; [doi: 10.1007/s11145-004-5894-0]
6. Dennis LR, Horn E. Strategies for Supporting Early Literacy Development. Young Except Child 2011; [doi: 10.1177/1096250611420553]
7. Hay I, Fielding-Barnsley R. Competencies that underpin children’s transition into early literacy. Aust J Lang Lit 2009; PMID:175715
8. Massetti GM, Bracken SS. Classroom academic and social context: Relationships among emergent literacy, behavioural functioning and teacher curriculum goals in kindergarten. Early Child Dev Care 2010; [doi: 10.1080/03004430801917401]
9. Missall KN, McConnell SR, Cadigan K. Early literacy development: Skill growth and relations between classroom variables for preschool children. J Early Interv 2006; [doi: 10.1177/105381510602900101]
10. Rideout, V., & Robb, M. B. (2020). The Common Sense census: Media use by kids age zero to eight, 2020. San Francisco, CA: Common Sense Media
11. Radesky JS, Schumacher J, Zuckerman B. Mobile and Interactive Media Use by Young Children: The Good, the Bad, and the Unknown. Pediatrics 2015; [doi: 10.1542/peds.2014-2251]
12. Parish-Morris J, Mahajan N, Hirsh-Pasek K, Golinkoff RM, Collins MF. Once upon a time: Parent-child dialogue and storybook reading in the electronic era. Mind, Brain, Educ 2013; [doi: 10.1111/mbe.12028]
13. Ambrose SE, VanDam M, Moeller MP. Linguistic input, electronic media, and communication outcomes of toddlers with hearing loss. Ear Hear. 2014 Mar-Apr;35(2):139-47. doi: 10.1097/AUD.0b013e3182a76768. PMID: 24441740; PMCID: PMC3944057.
14. Gaffney, M., et al. "Identifying infants with hearing loss-United States, 1999-2007." Morbidity and mortality weekly report 59.8 (2010): 220-223
15. *Ching TYC, Dillon H, Leigh G, Cupples L. Learning from the Longitudinal Outcomes of Children with Hearing Impairment (LOCHI) study: summary of 5-year findings and implications. Int J Audiol. 2018 May;57(sup2):S105-S111. doi: 10.1080/14992027.2017.1385865. Epub 2017 Oct 12. PMID: 29020839; PMCID: PMC5897193.*
16. Niparko JK, Tobey EA, Thal DJ, Eisenberg LS, Wang NY, Quittner AL, Fink NE; CDaCI Investigative Team. Spoken language development in children following cochlear implantation. JAMA. 2010 Apr 21;303(15):1498-506. doi: 10.1001/jama.2010.451. PMID: 20407059; PMCID: PMC3073449.
17. Geers AE, Hayes H. Reading, writing, and phonological processing skills of adolescents with 10 or more years of cochlear implant experience. Ear Hear. 2011 Feb;32(1 Suppl):49S-59S. doi: 10.1097/AUD.0b013e3181fa41fa. PMID: 21258612; PMCID: PMC3023978.
18. Mayer C, Trezek BJ. Literacy Outcomes in Deaf Students with Cochlear Implants: Current State of the Knowledge. J Deaf Stud Deaf Educ. 2018 Jan 1;23(1):1-16. doi: 10.1093/deafed/enx043. PMID: 29040702.
19. Stacey L. Children with hearing loss: developing listening and talking, birth to six, 3rd edition. Int J Audiol 2017; [doi: 10.1080/14992027.2016.1270470]
20. Percy-Smith L, Tønning TL, Josvassen JL, Mikkelsen JH, Nissen L, Dieleman E, Hallstrøm M, Cayé-Thomasen P. Auditory verbal habilitation is associated with improved outcome for children with cochlear implant. Cochlear Implants Int. 2018 Jan;19(1):38-45. doi: 10.1080/14670100.2017.1389020. Epub 2017 Oct 23. PMID: 29058555.
21. Bus AG, van IJzendoorn MH, Pellegrini AD. Joint Book Reading Makes for Success in Learning to Read: A Meta-Analysis on Intergenerational Transmission of Literacy. Rev Educ Res 2008; [doi: 10.3102/00346543065001001]
22. Mol SE, Bus AG, de Jong MT. Interactive Book Reading in Early Education: A Tool to Stimulate Print Knowledge as Well as Oral Language. Rev Educ Res 2009; [doi: 10.3102/0034654309332561]
23. Van Kleeck A, Woude J Vander. Book sharing with preschoolers with language delays. Read Books to Child Parents Teach 2003. [doi: 10.4324/9781410607355]
24. NAEYC. EARLY LEARNING STANDARDS: CREATING THE CONDITIONS FOR SUCCESS. 2002.
25. Lonigan, Christopher & Shanahan, Timothy. (2009). Developing Early Literacy: Report of the National Early Literacy Panel. Executive Summary. A Scientific Synthesis of Early Literacy Development and Implications for Intervention. National Institute for Literacy. http://www.nifl.gov/earlychildhood/NELP/NELPreport.html
26. Whitehurst, G. J., Falco, F. L., Lonigan, C., Fischel, J. E., DeBaryshe, B. D., & Valdez-Menchaca, M. C., et al. (1988). Accelerating language development through picturebook reading. Developmental Psychology, 24, 552–558
27. Mol, S. E., Bus, A. G., de Jong, M. T., & Smeets, D. J. (2008). Added value of dialogic parent-child book readings: A meta-analysis: Early Education and Development, 19, 7–26. doi:10.1080/1040928070183603.
28. Hindman AH, Wasik BA. Vocabulary learning in Head Start: Nature and extent of classroom instruction and its contributions to children's learning. J Sch Psychol. 2013 Jun;51(3):387-405. doi: 10.1016/j.jsp.2013.01.001.. PMID: 23816231.
29. DesJardin JL, Doll ER, Stika CJ, Eisenberg LS, Johnson KJ, Ganguly DH, Colson BG, Henning SC. Parental Support for Language Development During Joint Book Reading for Young Children With Hearing Loss. Commun Disord Q. 2014 May;35(3):167-181. doi: 10.1177/1525740113518062. PMID: 25309136; PMCID: PMC4191727.
30. Fung PC, Chow BW, McBride-Chang C. The impact of a dialogic reading program on deaf and hard-of-hearing kindergarten and early primary school-aged students in Hong Kong. J Deaf Stud Deaf Educ. 2005 Winter;10(1):82-95. doi: 10.1093/deafed/eni005. PMID: 15585750.
31. Trussell JW, Easterbrooks SR. The effect of enhanced storybook interaction on signing deaf children's vocabulary. J Deaf Stud Deaf Educ. 2014 Jul;19(3):319-32. doi: 10.1093/deafed/ent055. Epub 2013 Dec 16. PMID: 24344161.
32. Sherri Fickenscher & Dan Salvucci. Chapter 7 Listening & Spoken Language Strategies. An Introduction to Educating Children Who are Deaf/Hard of Hearing https://www.infanthearing.org/ebook-educating-children-dhh/chapters/7%20Chapter%207%202020.pdf
33. Leybaert J, LaSasso CJ. Cued speech for enhancing speech perception and first language development of children with cochlear implants. Trends Amplif. 2010 Jun;14(2):96-112. doi: 10.1177/1084713810375567. PMID: 20724357; PMCID: PMC4111351
34. Dorman MF, Liss J, Wang S, Berisha V, Ludwig C, Natale SC. Experiments on Auditory-Visual Perception of Sentences by Users of Unilateral, Bimodal, and Bilateral Cochlear Implants. J Speech Lang Hear Res. 2016 Dec 1;59(6):1505-1519. doi: 10.1044/2016_JSLHR-H-15-0312. PMID: 27960006; PMCID: PMC5399766.
35. Schorr EA, Fox NA, van Wassenhove V, Knudsen EI. Auditory-visual fusion in speech perception in children with cochlear implants. Proc Natl Acad Sci U S A. 2005 Dec 20;102(51):18748-50. doi: 10.1073/pnas.0508862102. Epub 2005 Dec 8. PMID: 16339316; PMCID: PMC1317952.
36. Abu-Zhaya R, Kondaurova MV, Houston D, Seidl A. Vocal and Tactile Input to Children Who Are Deaf or Hard of Hearing. J Speech Lang Hear Res. 2019 Jul 15;62(7):2372-2385. doi: 10.1044/2019_JSLHR-L-18-0185. Epub 2019 Jun 27. PMID: 31251677; PMCID: PMC7251336.
37. Brackenbury T, Ryan T, Messenheimer T. Incidental word learning in a hearing child of deaf adults. J Deaf Stud Deaf Educ. 2006 Winter;11(1):76-93. doi: 10.1093/deafed/enj018. Epub 2005 Nov 17. PMID: 16293808.
38. Easterbrooks, S. R., & Baker, S. K. (2002). Language learning in children who are deaf and hard of hearing: Multiple pathways. Boston, MA: Allyn & Bacon.
39. Nittrouer S. Early development of children with hearing loss. San Diego: Plural Publishing; 2010.
40. Lund E. Vocabulary Knowledge of Children With Cochlear Implants: A Meta-Analysis. J Deaf Stud Deaf Educ. 2016 Apr;21(2):107-21. doi: 10.1093/deafed/env060. Epub 2015 Dec 27. PMID: 26712811; PMCID: PMC4886318.
41. Messier J, Wood C. Facilitating Vocabulary Acquisition of Children With Cochlear Implants Using Electronic Storybooks. J Deaf Stud Deaf Educ. 2015 Oct;20(4):356-73. doi: 10.1093/deafed/env031. Epub 2015 Aug 6. PMID: 26251346.
42. Luckner JL, Cooke C. A summary of the vocabulary research with students who are deaf or hard of hearing. Am Ann Deaf. 2010 Spring;155(1):38-67. doi: 10.1353/aad.0.0129. PMID: 20503907.
43. Miller, E. M., Lederberg, A. R., & Easterbrooks, S. R. (2013). Phonological awareness: Explicit instruction for young deaf and head-of-hearing children. Journal of Deaf Studies and Deaf Education, 18, 206–227. doi:10.1093/deafed/ens067
44. Hirsh-Pasek K, Zosh JM, Golinkoff RM, Gray JH, Robb MB, Kaufman J. Putting Education in “Educational” Apps: Lessons From the Science of Learning. Psychol Sci Public Interes Suppl 2015; [doi: 10.1177/1529100615569721]
45. Beal-Alvarez, J. S., & Cannon, J. E. (2014). Technology intervention research with Deaf and hard of hearing learners: Levels of evidence. American Annals of the Deaf, 158, 486–505. doi:10.1353/aad.2014.0002
46. DeForte S, Sezgin E, Huefner J, Lucius S, Luna J, Satyapriya AA, Malhotra P. Usability of a Mobile App for Improving Literacy in Children With Hearing Impairment: Focus Group Study. JMIR Hum Factors. 2020 May 28;7(2):e16310. doi: 10.2196/16310. PMID: 32205305; PMCID: PMC7290449.
