# Supplementary material for: Study protocol for Hear Me Read (HMR): A prospective clinical trial assessing a digital storybook intervention for young children who are deaf or hard of hearing
Source: PLoS One. 2024 May 31;19(5):e0302734. doi: 10.1371/journal.pone.0302734 (PMC11142545; doi:10.1371/journal.pone.0302734)
Supplement: S3 File — (DOCX) [file pone.0302734.s003.docx]

## CONSENT TO PARTICIPATE IN A CLINICAL RESEARCH STUDY

**STUDY TITLE: Effectiveness of adding a novel digital storybook intervention platform in therapy to improve speech, language, and literacy outcomes in children with hearing loss**

**PRINCIPAL INVESTIGATOR: Prasanth Pattisapu, MD**

**TELEPHONE NUMBER: 000-000-0000**

**PARTICIPANT’S NAME: DATE OF BIRTH:**

**CHILD’S NAME: DATE OF BIRTH:**

**NOTE: The words “you” and “your” are used in this consent form. These words refer to the study volunteer whether a child or an adult*.***

**Key Information About This Study**

The following is a short summary of this study to help you decide whether or not to participate. More detailed information follows later in this form.

The purpose of this study is to find out if an iPad application (“app”) called Hear Me Read, can help children with hearing loss gain more from speech therapy. The study team has developed the app for children with hearing loss, their parent/guardian(s), and clinicians (speech language pathologists (SLPs)).

Study participation: Each family is invited to take part in 2 separate 6-month intervention periods, for a total of 12 months of participation. During both intervention periods, your child will continue to see their SLP as they normally would. In-person therapy frequency is decided by your child’s treating SLP and may range from monthly to weekly. During the second intervention period, we ask that you and your child also use the Hear Me Read app.

We will ask you to complete 3 surveys during the 12 months. Once at the beginning (0 months), once in the middle (6 months) and once at the end of the study (12 months). The surveys will ask about you, your family, and your experiences with speech therapy and the app. You can answer these questions at your home, at a time that is convenient for you. We may also ask you to periodically complete a brief reading questionnaire to learn about your reading habits.

Study visits: You will have 3 meetings with research staff. These can be done in-person or virtually. In- person research activities will be coordinated with standard of clinical care visits. You do not have to come to Nationwide Children’s Hospital outside of when you usually come for your child’s speech therapy and other clinical appointments to participate in this study.

The main risk of the study is more time spent using screens/digital devices. To minimize this, we recommend following the guidelines by the American Academy of Pediatrics for safe media use in children. We will provide you with this information today. The time spent using the Hear Me Read app in this study is not more than the recommendations. Other risks are listed later in this form.

There are no direct benefits to study participants. However, we hope to use the information and feedback provided to improve this app. Another potential benefit is increased reading and book engagement which generally provide educational and developmental benefits in all children.

If you are interested in learning more about this study, please continue reading below.

## INTRODUCTION

We invite you and your child to be part of this research study. Using this form as a guide, we will explain the study to you. If you have any questions about the study, please ask. By signing this form, you agree that you and your child can take part in this study. If you do not want your child to be in this study, all regular and standard medical care will still be available to your child here at Nationwide Children’s Hospital. Participation is voluntary. You and your child can leave this study at any time.

You will be given or emailed a signed and dated copy of the consent form.

## WHERE WILL THE STUDY BE DONE AND HOW MANY SUBJECTS WILL TAKE PART?

This study will be done at Nationwide Children’s Hospital and we hope to enroll 50 caregivers and their child. The study will also enroll Speech Language Pathologists for each of the 50 children.

## WHAT WILL HAPPEN DURING THE STUDY AND HOW LONG WILL IT LAST?

You and your child will be enrolled in this study for 12 months. If you sign up, you will meet with research staff three times. These meetings can be done in-person or virtually. Your child will continue to attend their speech therapy appointments as they usually would.

There are *two intervention periods* in this study. Each one lasts about 6 months. Every participant will participate in both intervention periods.

## Intervention Period 1 (SLT)

- This is a Speech and Language Therapy-only Intervention (SLT). Your child will go to speech therapy as they typically would. This is not different than the speech therapy your child would receive if you were not participating in the study. This happens for the first 6 months of the study.
- Standardized reading time will be prescribed by your child’s SLP at 20 minutes, 3 times a week. This reading time will be completed by you and your child at home.
- You or your child’s SLP will fill out a brief reading questionnaire at least monthly to collect information about caregiver-reported reading habits.

## Intervention Period 2 (SLT+Digital)

- The second part of the study is the Speech-Language Therapy with the Hear Me Read Intervention (SLT+Digital). Your child will go to speech therapy as they typically would. This is not different than the speech therapy your child would receive if you were not participating in the study. We will also ask you and your child to use the use the Hear Me Read app. This happens during the last 6 months of the study.
- Standardized reading time will be prescribed by your child’s SLP at 20 minutes, 3 times a week. This reading time will be completed by you and your child at home.
- Hear Me Read is an app that enables caregivers and SLPs to partner together to help children who are deaf or hard of hearing achieve reading, speech, and language goals through interactive digital storybook reading.
- We will provide you with a study tablet to use during this part of the study.
- Hear Me Read storybooks will be completed during your child’s in-person therapy sessions. Your therapist will assign storybooks in Hear Me Read for you and your family to read at home with your child.
- You or your child’s SLP will fill out a brief reading questionnaire at least monthly to collect caregiver-reported reading habits.

Over the 12 months of the study, there are *3 meetings with research staff*. These meetings can happen in-person before or after your speech therapy appointment or we can do them virtually.

## Meeting 1: Enrollment and Pre-Intervention Survey (in-person and/or virtual), Month 0

- You will be given a study packet which includes information about the study as well as recommendations from American Academy of Pediatrics for safe media use in children.
- We will ask you to complete questionnaires about you and your child and your reading habits. These questions will take about 5-10 minutes to answer.

## Meeting 2: Post SLT Intervention (in-person and/or virtual), Month 6

- You will be given a study packet which includes information about Hear Me Read such as instructions and troubleshooting information. We will give you contact information of someone to contact if the app isn’t working.
- We will assign a study iPad with Hear Me Read installed for your family to use during the study.
- We will ask you to answer questions about your experiences in speech therapy. These questions will take about 5 minutes to answer.
- We will ask your child’s SLP to answer similar questions.

## Meeting 3: Post SLT+Digital Intervention (in-person and/or virtual), Month 12

- We will ask you to return the study iPad that we gave you at Meeting 2.
- We will ask you to answer questions about your experiences in speech therapy. These questions will take about 5 minutes to answer.
- We will also ask you questions about the Hear Me Read app and how it worked for you. These questions will take about 10-15 minutes to answer.
- We will ask your child’s SLP to answer similar questions.

In addition to the research activities above, your child will participate in their speech therapy sessions, just as they would if they did not participate in the study. We will gather information about these sessions (e.g., speech, language, and literacy outcome measures), your child’s hearing loss, and other relevant factors (e.g., demographics, mode of communication) from your child’s medical record.

We also ask your child’s SLP to record audio and video of your child’s speech-language therapy evaluations while you are in the study. A secure NCH device will be used to record the evaluation. This will help our team learn about your child’s speech and language outcomes while they are in the Hear Me Read study. The recordings will be stored on a secure Nationwide Children’s drive accessible only to study staff and your child’s SLP.

Your information will be used only for research and will not be sold. You will not receive any money or other compensation for any new products that might be developed or sold from this research.

This study will use Twilio, a third-party service, to send text messages to you. This text message will include a link. When you click the link, you will be directed to a secure site to complete study surveys. The text messages will not include PHI and/or Personally Identifiable Information (PII). The messages will come from the phone number 000-000-0000.

Because this is a third-party service, text messages will be routed through Twilio’s systems. NCH has policies and procedures to make sure that information is deleted from Twilio’s logs shortly after the message is sent.

Message/data rates may apply to the text messages. We are not responsible for any text-message related charges. If you do not want to communicate with the study team via text message or would like to stop receiving text messages at any time, please contact the study team via phone or email.

## WHAT ARE THE RISKS OF BEING IN THIS STUDY?

We believe that there is very little chance that bad things will happen as a result of being in this study.

It is possible that you could feel upset when answering questions about your child’s diagnosis or medical treatment, but it may be more likely that you find the questions or feedback process a little boring. If you do find any of the questions upsetting or don’t want to answer a question, you don’t have to, and the study team will be available to discuss this with you further.

The duration of time spent using screens/digital devices is important to consider. The prescribed reading time using the Hear Me Read app is not more than the recommendations made by the American Academy of Pediatrics, and we are not enrolling children aged 2 or younger (who are at greatest risk). See brochure **“Beyond Screen Time – A Parent’s Guide to Media Use”.**

Economic risks are not expected to be excessive, given that interactions will take place during standard clinical care visits. No additional parking or transportation needs are expected. Time spent learning and troubleshooting the software could occur. We will provide contact information to the Research Information Solutions and Innovation (RISI), the technology team that helped create the app.

This study involves questions or surveys that may make you feel depressed. You should call the Principal Investigator at 000-000-0000 if you notice any changes in your mood, ideas, or behavior.

Immediate help is available if thoughts or feelings of hurting oneself come up**.** Call one of the numbers below or go to the closest Emergency Room.

## Nationwide Children’s Psychiatric Emergency Evaluation Center 000-000-0000 Hopeline/Lifeline – 000-000-0000 or 000-000-0000

Although we will take every precaution, there is a small chance of loss of confidentiality of your study information. The study staff, and Nationwide Children’s Hospital technology teams, have taken precautions to ensure all the information, including videos, collected on the iPad are stored and transferred securely.

There may be other risks of being in this research study that are not known at this time.

## ARE THERE BENEFITS TO TAKING PART IN THIS STUDY?

Although there may be no benefit to your child from being in this study, we hope to learn something that could help other families of children who are hard of hearing.

Increased reading and book engagement may occur, and this generally provides educational and developmental benefits in all children, including those that are deaf or hard of hearing.

## WHAT ARE THE COSTS AND REIMBURSEMENTS?

All costs related to the research parts of this study will be covered by the research team. However, the parts of the study that would be done for routine clinical care will be billed to you and to your insurance company or third party payer. You may have to pay any costs that the insurance company or third party payer does not pay. The study team will discuss these costs with you, if applicable.

For your time and inconvenience, you will receive $10 per survey; up to a total of $30. You will be issued a debit card specially designed for clinical research. When a study survey is completed, funds will be approved and automatically loaded onto your card.

## WHAT HAPPENS IF BEING IN THIS STUDY CAUSES INJURIES?

We believe that there is very little chance that injuries will happen as a result of participating in this study.

## WHAT HAPPENS IF I DO NOT FINISH THIS STUDY?

It is your choice to participate in this study. You may decide to stop your participation at any time. If you decide to stop, call the study team at the number on page 1 of this form to see if there are any medical issues related to stopping. If you stop being in the study, there will be no penalty or loss of benefits to which you are otherwise entitled.

If at any time the Principal Investigator believes that this study is not good for you, the study team will contact you about stopping. If the study instructions are not followed, participation in the study may also be stopped. If unexpected medical problems come up, the Principal Investigator may decide to stop your participation in the study.

## OTHER IMPORTANT INFORMATION

If you are an employee of Nationwide Children’s Hospital or the Research Institute at Nationwide Children’s Hospital, your job or performance appraisal will not be affected in any way if you decline to participate or withdraw your consent to participate in this study.

A description of this clinical trial will be available on [http://www.ClinicalTrials.gov,](http://www.clinicaltrials.gov/) as required by

U.S. Law. This Website will not include information that can identify you. At most, the Website will include a summary of the results. You can search this Website at any time.

Speech, language, and literacy outcome measures that are collected as part of routine clinical care will be reported to the family, as part of standard care by the therapists. The final study results will not be shared with you individually. However, at some time, a final study summary will be available on the ClinicalTrials.Gov (http://clinicaltrials.gov) website.

A company called "Digital Story Therapies, Inc." paid money to Nationwide Children's Hospital (NCH) to license the Hear Me Read app. NCH may receive money in the future from Digital Story Therapies, Inc. for information from the app and if the app is sold. NCH will share some money with Dr. Prashant Malhotra. Dr. Malhotra is a consultant in the Hear Me Read study. He is also the founder and part-owner of Digital Story Therapies, Inc. Dr. Malhotra was the person leading the research project. This is also known as the Principal Investigator (PI). Because he is the founder and part-owner of Digital Story Therapies, Inc., he is no longer leading the project. The PI is now Dr. Prasanth Pattisapu.

If you have any questions about this study after reading this information, please contact the PI, Prasanth Pattisapu at 000-000-0000 or [Prasanth.Pattisapu@NationwideChildrens.org](mailto:Prasanth.Pattisapu@NationwideChildrens.org), or the Study Coordinator, Chenelle Miller at 000-000-0000 or [Chenelle.Miller@NationwideChildrens.org](mailto:Chenelle.Miller@NationwideChildrens.org).

If you are not comfortable talking with the study team, you can contact the Office of Compliance and Integrity via email at [ComplianceIntegrity@nationwidechildrens.org](mailto:ComplianceIntegrity@nationwidechildrens.org) or phone at 000-000-0000. You may also call the Anonymous Hotline at 000-000-0000.

Nationwide Children’s Hospital is a teaching hospital, and we are committed to doing research. Conducting research will enable us to learn and provide the best care for our patients and families. You may be asked to participate in other research studies in the future. You have the right to decide to participate or decline to participate in any future studies. We will not share your contact information with researchers outside Nationwide Children’s Hospital.

## HOW WILL MY STUDY INFORMATION BE KEPT PRIVATE?

Information collected for this study includes information that can identify you. This is called “protected health information” or PHI. By agreeing to be in this study, you are giving permission to your health care provider to use or disclose (release) your health information that identifies you for the research study described in this form. Information collected is the property of Nationwide Children’s Hospital, its affiliated entities, and/or the sponsor*.*

PHI that may be used or disclosed will include:

- Name
- Complete Address
- Telephone or Fax Number
- Dates (treatment dates, birth date, date of death)
- Email address, IP address or url
- Medical Record Number
- Full face photography (videos)
- Biometric identifiers, including voice prints

## People or Companies authorized to use, disclose, and receive PHI collected or created by this research study:

- - PI and Study Staff
  - The Nationwide Children’s Hospital Institutional Review Board (the committee that reviews all human subject research)
  - Nationwide Children’s Hospital internal auditors
  - Your insurance company

Because of the need to give information to these people, absolute confidentiality cannot be guaranteed. Information given to these people may be further disclosed by them and no longer be protected by federal privacy rules.

**Reason(s) why the use or disclosure is being made:** Name, complete address, email address, date of birth, medical record number and phone number will be used to locate medical charts and to contact you. Treatment dates will be used to provide information about your child’s hearing loss. Video and audio recordings will be used in the Hear Me Read app and to record your child’s standard of care speech therapy assessments.

You may decide not to authorize the use and disclosure of your PHI. However, if it is needed for this study, you will not be able to participate in this study. If you agree to be in this study and later decide to withdraw your participation, you may withdraw your authorization to use your PHI. This request must be made in writing to the Principal Investigator at:

Attn: Prasanth Pattisapu, MD

Suite T2G

Nationwide Children's Hospital Columbus, OH 43205

If you withdraw your authorization, no new PHI may be collected and the PHI already collected may not be used unless it has already been used or is needed to complete the study analysis and reports.

PHI will only be shared with the groups listed above, but if you experience a bad outcome or adverse event from being in this study, the study team or other health care providers may need to look at your entire medical records.

The results from this study may be published but your identity will not be revealed.

A copy of this form and other research related health information may be added to your NCH medical record.

The PHI collected or created under this research study will be used or disclosed as needed until the end of the study. The records of this study will be kept for an indefinite period of time and your authorization to use or disclose your PHI will not expire.

## USE OF INFORMATION/SAMPLES FOR FUTURE RESEARCH USE Future Research Use of Identifiable Information:

With your permission, we would like to store your identifiable information (including PHI) for future research purposes, and as part of such future research purposes, your identifiable information may be disclosed to people or entities not listed above, such as researchers not involved with this study, government agencies, research foundations, or pharmaceutical or device companies. This future research may or may not be related to your medical problem. This future research may include sensitive information. Any future research projects will be reviewed and approved by an Institutional Review Board which protects the rights, welfare, and safety of human research subjects. If your identifiable information including PHI is used or disclosed in future research studies, absolute confidentiality cannot be guaranteed. Information shared for future research may be shared further with others and no longer be protected by federal privacy rules.

If you decide at any time that you do not want your PHI stored for future research, you must make this request in writing to the Principal Investigator at*:*

Attn: Prasanth Pattisapu, MD

Suite T2G

Nationwide Children's Hospital Columbus, OH 43205

Once we receive your written request, we will destroy your PHI. However, if we have already shared your PHI with another individual or entity, we will not be able to destroy any of the PHI that are no longer in our possession.

Nationwide Children’s Hospital retains the right to cease storage and destroy the PHI at any time without sending notice to you or obtaining your consent.

You do not have to agree to use of your PHI for future research in order to participate in this study, and your decision will not affect the care you receive from the study doctors or Nationwide Children’s Hospital.

I agree to allow my PHI to be stored and used for future research as described above: (initial your choice)

YES _____ NO

## WHOM SHOULD I CALL IF I HAVE QUESTIONS OR PROBLEMS?

If you have any study-related questions during your participation or you have been injured by the research, you may contact the Principal Investigator at 000-000-0000, Monday – Friday, between *8:30am-4:30pm*.

If you have questions, concerns, or complaints about the research; if you have questions about your rights as a research volunteer; if you cannot reach the Principal Investigator; or if you want to call someone else, call 000-000-0000, Nationwide Children's Hospital Institutional Review Board, (the committee that reviews all research involving human subjects at Nationwide Children’s Hospital).

# Signature Block for Children N/A, Adult Subject

Your signature documents your permission for the named child to take part in this research.

Printed name of child

Signature of parent or individual legally authorized to consent Date & Time AM/PM

to the child’s general medical care

Printed name of parent or individual legally authorized to consent

to the child’s general medical care

Relationship to Participant

Note: Investigators are to ensure that individuals who are not parents can demonstrate their legal authority to consent to the child’s general medical care. Contact Legal Services if any questions arise.

Signature of second parent or individual legally authorized to Date & Time AM/PM

consent to the child’s general medical care

Printed name of second parent or individual legally authorized to

consent to the child’s general medical care

Relationship to Participant

If signature of second parent not obtained, indicate why: (select one)

- Not required by IRB
- Second parent is deceased
- Second parent is unknown
- Second parent is incompetent
- Second parent is not reasonably available
- Only one parent has legal responsibility for the care and custody of the child

Signature of person obtaining consent Date & Time AM/PM

Printed name of person obtaining consent

# Signature Block for Adult Participation N/A, Pediatric Subject

Your signature documents your permission to take part in this research.

Signature of subject Date & Time AM/PM

Printed name of subject

Signature of person obtaining consent Date & Time AM/PM

Printed name of person obtaining consent

# Signature Block for Adult Unable to Consent N/A

Your signature documents your permission for the named person to take part in this research.

Printed name of subject

Signature of legally authorized representative Date & Time AM/PM

Printed name of legally authorized representative

Relationship to Participant

| Signature of person obtaining consent |  |  | Date & Time | AM/PM |
| --- | --- | --- | --- | --- |
| Printed name of person obtaining consent  **Assent** |  |  |  |  |
| Signature of subject |  |  | Date & Time | AM/PM |

- Not obtained because the capability of the subject is so limited that the subject cannot reasonably be consulted.
