# Supplementary material for: Study protocol for Hear Me Read (HMR): A prospective clinical trial assessing a digital storybook intervention for young children who are deaf or hard of hearing
Source: PLoS One. 2024 May 31;19(5):e0302734. doi: 10.1371/journal.pone.0302734 (PMC11142545; doi:10.1371/journal.pone.0302734)
Supplement: S1 Table — (DOCX) [file pone.0302734.s004.docx]

| Clinical Evaluation of Language Fundamentals Preschool-3 Subtests and Indices [[22](https://clinicaltrials.gov/ct2/show/study/NCT05245799?term=hear+me+read&draw=2&rank=1)]  *Higher scores indicating better *language and literacy* outcomes | |
| --- | --- |
| **Language** | |
| Word Structure (WS) | Evaluate examinee’s ability to (1) apply word structure rules to mark inflections, derivations, and comparison and (2) select and use appropriate pronouns to refer to people , objects, and possessive relationships. ***Raw scores (min = 0; max = 24)*** are reported as standard scores, percentile ranks, scaled scores, and age equivalents. |
| Sentence Comprehension (SC) | Evaluate examinee’s ability to interpret spoken sentences of increasing length and complexity (i.e., examinee identifies the picture that matches the sentence read aloud by examiner). ***Raw scores (min = 0; max = 22)*** are reported as standard scores, percentile ranks, scaled scores, and age equivalents. |
| Expressive Vocabulary (EV) | Evaluate examinee’s ability to label images of people, objects, and actions. ***Raw scores (min = 0; max = 42)*** are reported as standard scores, percentile ranks, scaled scores, and age equivalents. |
| Following Directions (FD) | Evaluate examinee’s ability to (1) interpret spoken directions of increasing length and complexity, (2) remember the names, characteristics, and order of mention of pictures, and (3) identify the targets from among several choices. ***Raw scores (min = 0; max = 24)*** are reported as standard scores, percentile ranks, scaled scores, and age equivalents. |
| Recalling Sentences (RS) | Evaluate examinee’s ability to listen to spoken sentences of increasing length and complexity and repeat the sentences without changing word meanings, inflections, derivations or comparisons, or sentence structure. ***Raw scores (min = 0; max = 45)*** are reported as standard scores, percentile ranks, scaled scores, and age equivalents. |
| Basic Concepts (BC) | Evaluate examinee’s knowledge of concepts including direction/location/position, number/quantity, sequence, attributes, dimension/size, same/different, and inclusion/exclusion. ***Raw scores (min = 0; max = 24)*** are reported as standard scores, percentile ranks, scaled scores, and age equivalents. |
| Word Classes (WC) | Evaluate examinee’s ability to perceive relationships between words that are related by semantic class features. ***Raw scores (min = 0; max = 20)*** are reported as standard scores, percentile ranks, scaled scores, and age equivalents. |
| Core Language Score (CLS) | Measurement of general language ability. Scores are derived by summing the scaled scores from the subtests that best discriminate typical language performance from disordered language performance deriving a standardized ***composite score (min = 45; max = 155)***. |
| Receptive Language Index (RLI) | Measurement of listening and auditory comprehension. Scores are derived by summing the scaled scores from a combination of two or three receptive subtests revealing a standardized ***composite score (min = 45; max = 155)***. |
| Expressive Language Index (ELI-lan) | Measurement of performance on three subtests that probe expressive language. Scores are derived by summing the scaled scores from the subtests and deriving a standardized ***composite score (min = 45; max = 155)***. |
| **Literacy** | |
| Pre-literacy Rating Scale (PRS) | Identifies preliteracy skills that may influence development of reading and writing skills based on information from an informant about the examinee’s early reading and writing skills. ***Raw scores (min = 0; max = 105)*** are reported as standard scores, percentile ranks, scaled scores, and age equivalents. |
| Phonological Awareness (PA) | Evaluate examinee’s knowledge of the sound structure of language and ability to manipulate sound through compound word and syllable blending, sentence and syllable segmentation, and rhyme awareness and production. ***Raw scores (min = 0; max = 24)*** are reported as standard scores, percentile ranks, scaled scores, and age equivalents. |
| Early Literacy Index (ELI-lit) | Measurement of performance on the PRS and PA subtests designed to access early literacy skills. Scores are derived by summing the scaled scores from the subtests and deriving a standardized ***composite score (min = 45; max = 155)***. |
