# Supplementary material for: Study protocol for Hear Me Read (HMR): A prospective clinical trial assessing a digital storybook intervention for young children who are deaf or hard of hearing
Source: PLoS One. 2024 May 31;19(5):e0302734. doi: 10.1371/journal.pone.0302734 (PMC11142545; doi:10.1371/journal.pone.0302734)
Supplement: Attachment — Submitted filename: Response to Reviewers.docx [file pone.0302734.s005.docx]

**Reviewer 1 Comments to the Author:**

Comment: This particular pre-post design is suitable when one assumes that the underlying mechanism, in this case, the improvement in children's speech and language skills, remains relatively consistent or stable over time. It is reasonable to assume that providing standard therapy over the 12-month period alone should lead to some level of improvement in the measured outcomes. If that is indeed the case, the difference between the two conditions (SLT-only during the first six months and SLT+Digital during the last six months) will encompass both the impact of 'natural progress' (i.e., improvement resulting from standard treatment without digital augmentation) and the progress due to the addition of the digital intervention. In essence, these two effects are intertwined and challenging to distinguish from one another.
A potentially better approach could involve a parallel group design, where children are randomly assigned to one of the two interventions, eliminating the confounding of effects, and allowing for a clearer assessment of the digital intervention's true impact.

Response: Thank you for your comment. A parallel group design insinuates that children would be randomized into one of two groups (clinical care as usual or clinical care + HMR intervention). While this would be ideal, this design does not account for the differences in therapy frequency. These differences range from weekly in-person speech therapy to evaluation-only speech therapy every six months. Additionally, this is a pilot trial with a small sample size (N=50). Using a within-subjects design allows us to achieve greater statistical power despite our small sample. Lastly, children act as their own control, which decreases variability (e.g., individual differences in therapy frequency and patient characteristics) and allows for greater confidence that changes in outcome measures are due to the addition of the Hear Me Read intervention.

Regarding natural progress: The CELF-P3 and ROWPVT-4 are standardized, norm-referenced, speech-language assessments.

Before enrollment, children complete their Pre-Trial evaluation. About six months later, they complete the SLT-only evaluation. Lastly, they complete the SLT+Digital evaluation around 12 months of participation. Children only receive clinical recommendations (i.e., speech therapy, reading, and literacy recommendations from the speech therapist) from the Pre-Trial to SLT only evaluation. We expect to understand the child’s natural progress within this period. From the SLT only to SLT+Digital evaluation, children receive clinical recommendations and the HMR application. Residual changes in scores are expected to be attributed to the HMR application. Additionally, changes in speech, language, and literacy for children who are D/HH are most sensitive to early reading. As such, we track reading progress (using the weekly reading questionnaires) and employ standardized reading time (20 minutes per day, three times per week) throughout the study duration.

**Reviewer 2 Comments to the Author:**

Comment 1: Statistical Analysis section: State the type of repeated measures analysis that will be used “to evaluate the effect of intervention on these outcomes.” (p. 13).

Response: A repeated measures approach using a mixed effects model will be used to compare the change across time in group 1 to the change across time in group 2 (SLT-Only to SLT+Digital) [see lines 288-291 in revised manuscript].

Comment 2: Statistical Analysis section: Explain how the primary and secondary outcome measures will be evaluated. Expand upon the statement: “For primary and secondary outcome measures, changes in raw score, standard score, percentile rank, scaled score, and age equivalent for the ROWPVT-4 and CELFP3 will be evaluated.”

Response: Changes in raw score are reported as standard score, percentile rank, scaled score, and age equivalent for the ROWPVT-4 and CELF-P3. These changes will be evaluated given the sensitivity of raw scores [see lines 291-293 in revised manuscript].

Comment 3: Identify the statistical method(s) that will be used to evaluate the patient characteristics associations with changes in outcomes.

Response: We will use t-tests and Wilcoxon-Mann-Whitney tests to identify patient characteristics associated with changes in outcomes [see lines 294-295 in revised manuscript].

Comment 4: 4- State how the data will be summarized: means, standard deviations, medians, interquartile range, frequencies, percentages, etc.

Response: A table in the final manuscript will provide baseline characteristics of participants. This will include means (SD), medians (IQR), and frequencies, as appropriate given the baseline variable of interest. Outcome measures will report the change in means from pre-trial to SLT only, from SLT only to SLT+Digital, and from pre-trial to SLT+Digital.

Comment 5: To assist in the review process, add line numbering to the document.

Response: Line numbers were added to the document.

**Reviewer 3 Comments to the Author:**

Comment: Overall, the article details the study plans and protocol in a way that is well-reasoned, structured, and informative. The authors make a strong case for the need for an intervention such as HMR and describe plans to trial it with a study employing rigorous methodology. The use of a repeated measures design and a within-subjects approach allows for a more accurate assessment of the impact of digital intervention by eliminating potential confounding variables. This approach certainly enhances the validity of your study findings. It will be interesting to see your study population and how you might consider or account for the potential for differences in growth between Time measure 1 (baseline to post-SLT-only) and Time measure 2 (post-SLT-only to post-SLP+digital) that could be due to child-specific or provider-specific variables in Time measure 2 (increased child age, increased child-provider rapport, increased time practicing therapy techniques, etc. You may wish to discuss in this or subsequent articles.

Response: Thank you for your comment. Child-, caregiver-, and provider-specific variables are important to consider. We will use t-tests and Wilcoxon-Mann-Whitney tests to identify patient characteristics associated with changes in outcomes. If any imbalance in patient characteristics (e.g., age, frequency of therapy) is detected between groups, the imbalanced characteristics will be adjusted for in a repeated measures approach using a mixed effects model [see lines 294-298 in the revised manuscript]. We expect parent/provider-specific variables to impact the child’s progress/outcomes equally during both study periods (Pre-Trial to SLT only and SLT only to SLT+Digital). Standard clinical care should minimize variability in how SLPs conduct speech therapy and administer speech evaluations. Regarding parent-specific variables, parents are instructed to read with their child 20 minutes per day, three times per week. Should differences in child-clinician rapport, parent availability to read with the child, etc., warrant investigating the effects of parent/provider-specific variables, we will discuss this in subsequent articles.

Comment: The regular follow-up with caregivers regarding book progress and completion of weekly reading questionnaires demonstrates a proactive approach to ensuring data accuracy and participant compliance. appreciated the included details about the features of the HMR application, such as the dual interface, personalized prompts, multimodal access to books, and encouragement of dialogic reading strategies were commendable. I would like to see more discussion related to the intervention design, such as how and why features and/or strategies were chosen to be part of the HMR intervention, including rationale from existing evidence, literature, other digital storybook interventions, etc. A description or examples of the expectations of use for participants would be helpful as well for readers to gain understanding of the application and the researchers’ intents for use as an effective intervention tool.

Response: Thank you for your comment. We conducted a preliminary usability assessment and employed qualitative interview techniques to understand the needs of children who are D/HH and their caregivers. This usability assessment found that families had default, specific, and family needs as it relates to the application. We developed the HMR application in line with these needs [see lines 204-215 in revised manuscript for more details].

Comment: While the article mentions that access to interim analyses and the dataset is restricted to authorized entities, it does not provide clear justification for this limitation. As written in the PLOS data policy, authors are required to make all data fully available as part of the manuscript or deposited to a public repository. The lack of public availability of the dataset hinders potential replication and further scrutiny of the study findings.

Response: Thank you for your comment. Given our small sample size (N=50), interim analyses are not planned. This prospective clinical trial will serve as pilot data to help us determine outcome measures for future trials. Anticipated completion of primary analyses is September 2024, at which point, result of outcome measures will be made available on ClinicalTrials.gov in line with their requirements. Additionally, see lines 299-300 in revised manuscript for updated language about data availability.
