# Supplementary material for: Study protocol for Hear Me Read (HMR): A prospective clinical trial assessing a digital storybook intervention for young children who are deaf or hard of hearing
Source: PLoS One. 2024 May 31;19(5):e0302734. doi: 10.1371/journal.pone.0302734 (PMC11142545; doi:10.1371/journal.pone.0302734)
Supplement: Attachment — Submitted filename: Response to Reviewers.docx [file pone.0302734.s006.docx]

**Journal Requirements:**

Please review your reference list to ensure that it is complete and correct. If you have cited papers that have been retracted, please include the rationale for doing so in the manuscript text, or remove these references and replace them with relevant current references. Any changes to the reference list should be mentioned in the rebuttal letter that accompanies your revised manuscript. If you need to cite a retracted article, indicate the article’s retracted status in the References list and also include a citation and full reference for the retraction notice.

Response: We have reviewed the reference list and identified no retracted articles. In response to Review 2 comments, we added the following to the reference list: (27) Kohn MA, Senyak J. Sample Size Calculators [website]. UCSF CTSI. 14 March 2024. Available at <https://www.sample-size.net/> [Accessed 19 March 2024].

**Reviewer 2 Comments to the Author:**

Comment 1: Line 220: Alpha levels rather than p-values are used in sample size calculations. Typically alpha levels are in the range of 0.01 to 0.10. Justify an alpha level of 0.001. It seems too small. Perhaps this is a typographical error.

Response 1: Thank you for pointing out this error. It appears that in addition to the typographical error of putting p instead of alpha, there was an error made in calculation by using alpha=0.001%, or 0.00001, instead of alpha=0.001. This caused the sample size estimate to be n=43, when the correct calculation of the given parameters gives n=27. This has been corrected in the submission [see lines 220-226, source cite in reference list (27)]. With regards to the alpha level, alpha=0.001 was chosen to be conservative and ensure that the treatment really was effective before expanding to larger studies, especially as HMR poses very low risk to patients. Using the same parameters but changing alpha level to 0.01 and 0.05 gives us sample sizes of n=19 and n=13, respectively, but for the purposes of our study we believe the feasibility assessment to be more important as we seek to gain as much information as possible.

Comment 2: Line 289: In the mixed effects model, it seems appropriate to first test the interaction of group by time.

Response 2: That is correct. We are comparing the changes between the two intervention periods (the SLT-Only and the SLT+Digital) which is a group by time interaction.

Comment 3: Typically continuous baseline characteristics/demographics are compared using t-tests or Wilcoxon rank sum tests and categorical data is compared between two groups using chi-square tests. T-tests and Wilcoxon Mann-Whitney U tests do not test for association, but differences between 2 groups.

Response 3: Thank you for your comment. We have included updated language as follows: “Baseline assessments are used to identify patient characteristics (e.g., age, frequency of therapy, rapport with SLP) associated with outcome variables using t-tests and correlation analyses.” [lines 293-295].

Comment 4: Line 295: The following statement is vague. Is the analytic approach mixed linear modeling? Clarify. "Characteristics associated with changes in the outcome (e.g., patient characteristics such as age, frequency of therapy) will be adjusted for within the context of the aforementioned analytic approach."

Response 4: We have clarified the statement as follows: “Characteristics associated with changes in the outcome (e.g., patient characteristics such as age, frequency of therapy) will be adjusted for within the context of a repeated measures approach using a mixed effects model” [see lines 296-298].
